# Mechanisms for Tuning Engineered Nanomaterials to Enhance Radiation Therapy of Cancer

**DOI:** 10.1002/advs.202003584

**Published:** 2020-10-28

**Authors:** Sandhya Clement, Jared M. Campbell, Wei Deng, Anna Guller, Saadia Nisar, Guozhen Liu, Brian C. Wilson, Ewa M. Goldys

**Affiliations:** ^1^ ARC Centre of Excellence for Nanoscale Biophotonics The Graduate School of Biomedical Engineering University of New South Wales High Street Kensington New South Wales 2052 Australia; ^2^ Institute for Regenerative Medicine Sechenov First Moscow State Medical University (Sechenov University) Trubetskaya Street Moscow 119991 Russia; ^3^ Department of Medical Biophysics University of Toronto/Princess Margaret Cancer Centre University Health Network Colledge Street Toronto Ontario ON M5G 2C1 Canada

**Keywords:** nanoparticles, radiation therapy, radiodynamic therapy, reactive oxygen species, X‐PDT

## Abstract

Engineered nanomaterials that produce reactive oxygen species on exposure to X‐ and gamma‐rays used in radiation therapy offer promise of novel cancer treatment strategies. Similar to photodynamic therapy but suitable for large and deep tumors, this new approach where nanomaterials acting as sensitizing agents are combined with clinical radiation can be effective at well‐tolerated low radiation doses. Suitably engineered nanomaterials can enhance cancer radiotherapy by increasing the tumor selectivity and decreasing side effects. Additionally, the nanomaterial platform offers therapeutically valuable functionalities, including molecular targeting, drug/gene delivery, and adaptive responses to trigger drug release. The potential of such nanomaterials to be combined with radiotherapy is widely recognized. In order for further breakthroughs to be made, and to facilitate clinical translation, the applicable principles and fundamentals should be articulated. This review focuses on mechanisms underpinning rational nanomaterial design to enhance radiation therapy, the understanding of which will enable novel ways to optimize its therapeutic efficacy. A roadmap for designing nanomaterials with optimized anticancer performance is also shown and the potential clinical significance and future translation are discussed.

## Graphical abstract

Engineered nanomaterials that produce reactive oxygen species on exposure to X‐ and gamma‐rays used in radiation therapy offer promise of novel cancer treatment strategies including in deep tissue. This review focuses on mechanisms underpinning rational nanomaterial design to enhance radiation therapy, the understanding of which will enable novel ways to optimize its therapeutic efficacy.

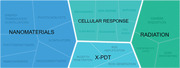

## 1 Introduction

Cancer is a complex disease whose treatment requires individually tailored modalities such as surgery, radiation therapy, chemotherapy, immunotherapy, and photodynamic therapy.^[^ ^1^, ^2^ ^]^ Many of these therapies have well‐known side effects, such as drug toxicity in the case of chemotherapy^[^ ^3^, ^4^ ^]^ and/or fibrosis or induction of late cancers in the case of radiation therapy.^[^ ^5^, ^6^ ^]^ Minimizing these off‐target effects represents an ongoing challenge.^[^ ^7^, ^8^, ^9^ ^]^ The potential for synergistic effects and dose reduction by combining treatment modalities, especially with radiotherapy that is used in ≈50% of cancer patients,^[^ ^10^ ^]^ has led to interest in combinational therapy approaches.^[^ ^11^ ^]^ These include radiosensitization, i.e., the use of various chemical agents that make cells more responsive to radiation therapy,^[^ ^12^ ^]^ aspects of which are discussed in this review. The efficacy of radiosensitizers is measured in terms of increased cancer cell kill while normal tissue function is retained. Thus, it is desirable that the radiosensitizers act differently in cancer and noncancer cells, rather than simply ensuring an overall amplification of the radiation dose.

Spatial and temporal control of treatment delivery is key in nonsurgical modalities of cancer treatment. Both radiotherapy, with its sophisticated treatment planning, and emerging modalities such as high intensity focussed ultrasound^[^ ^13^, ^14^ ^]^ provide excellent but not yet perfect spatial localization.^[^ ^15^, ^16^, ^17^ ^]^ Further improvement in tumor selectivity is promised by molecularly targeted nanoformulated agents^[^ ^18^, ^19^ ^]^ including those with enhanced functionalities.^[^ ^20^, ^21^ ^]^ Hence, combining radiotherapy and cancer‐targeted nanoparticle (NP) formulations is a logical step to more efficient cancer therapies, expected to offer expanded cancer treatment options and/or improved therapeutic efficacy. The uses of NPs in cancer treatment, including in radio‐oncology, have been reviewed previously,^[^ ^15^, ^22^, ^23^, ^24^, ^25^, ^26^, ^27^, ^28^, ^29^, ^30^, ^31^, ^32^, ^33^, ^34^, ^35^, ^36^ ^]^ while multimodal synergistic cancer therapies were extensively summarized elsewhere.^[^ ^11^, ^26^, ^27^, ^28^, ^29^, ^30^ ^]^ Significant caveats are that effective delivery of nanomaterials to tumor is still suboptimal and, ideally, treatment should not only destroy the primary tumor but also reduce the risk of recurrence. In addition, the use of such combined treatments must be aligned with clinical workflows.

X‐ray induced photodynamic therapy (X‐PDT) is a recently developed approach for cancer therapy which utilizes X‐ray as an energy source to activate reactive oxygen species (ROS) generation similarly to PDT where cytotoxicity is also due to the induction of ROS.^[^ ^27^, ^37^, ^38^ ^]^ Photodynamic therapy utilizes photosensitizer (PS) drugs that are designed to be activated by light in the presence of oxygen in the tissue to produce ROS that are cytotoxic. In X‐PDT, ROS are produced by X‐PDT agents that are activated by X‐rays instead of light, allowing much deeper tissue penetration of X‐PDT than traditional PDT. The X‐PDT agents investigated to‐date almost exclusively comprise specially designed NPs, although this is not a critical requirement. X‐PDT can be regarded as a unique radiosensitizing method, where radiosensitization occurs due to increased generation of reactive oxygen species.^[^ ^39^ ^]^ It is worth mentioning that some of the published literature uses the term radiodynamic therapy (RDT) referring to the situation where ionizing radiation (X‐ray) is used to excite a sensitizing agent.^[^ ^40^, ^41^, ^42^ ^]^ This term tends to be used when the sensitizing agent is a molecule. Here, we will use the term X‐PDT referring to the use of X‐rays to excite sensitizing agents regardless of their molecular or NP character (see Section 7 for more clarifications of terminology used in this field).

In this review, we focus on the rarely discussed physicochemical basis for X‐PDT radiosensitization,^[^ ^10^ ^]^ and the opportunities and limitations thereof, some of which draw on understanding of how ionizing radiation interacts with cells and tissues. In addition to DNA damage and antioxidant response, other cellular processes and mechanisms respond to radiation.^[^ ^12^ ^]^ These may offer suitable additional targets or bottlenecks that could be addressed by a suitable tailoring of the nanomaterials, and which may vary across cancer types. We further discuss the opportunities for optimizing the generation of cytotoxic free radicals by combining radiotherapy with tailored nanomaterials and consider the processes in cancer cells and tissues exposed to such combinatorial treatments including defence mechanisms. In discussing these topics, we identify the potential opportunities in materials science and some of the issues at the intersection of materials science and cell biology that are addressable by judicious nanomaterials engineering.

In order to limit the scope of this review, we do not cover issues related to NP delivery and tumor uptake, nor the processes that NP undergo in cells and organisms such as clearance, the effectiveness of the enhanced permeability and retention (EPR) effect on NP accumulation, and the stability of NPs in blood which have been expertly reviewed elsewhere.^[^ ^43^, ^44^, ^45^, ^46^, ^47^, ^48^, ^49^, ^50^ ^]^ Finally, we also do not discuss biological factors that impact cellular resilience to oxidative stress, or the intrinsic radiosensitivity of specific cell and tissue types^[^ ^51^ ^]^ that have also been reviewed.^[^ ^52^, ^53^ ^]^

**Figure 1:**
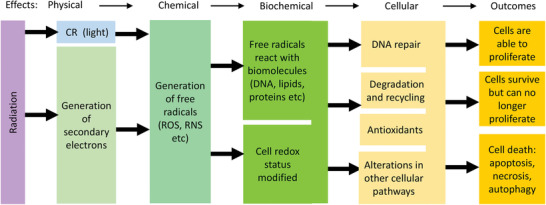
Illustration of physical, chemical, biochemical/biomolecular, and biological effects taking place in cells and tissues upon exposure to ionizing radiation. CR: Cherenkov radiation; ROS: reactive oxygen species; RNS: reactive nitrogen species.

The paper is organized as follows. In Sections 2 and 3 we discuss the cascade of physical, chemical, and biological events in cells and tissues exposed to ionizing radiation that affect their function, as illustrated in **Figure** **1**, and point to potential molecular targets for nanomaterials. Of specific interest are cellular systems of protection against oxidative stress that should be selectively disabled by prospective nanomaterial radiosensitizers. We focus on DNA damage and its repair, as well as on maintaining the redox status through the cellular antioxidant system. We also explore mechanisms of radiosensitization and ways that they can be modulated by radiosensitizers and exploited using designed nanomaterials.

**Figure 2:**
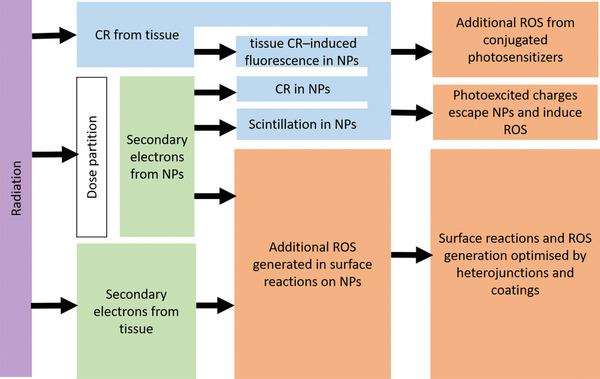
Interaction of radiation with NP constructs.

We then discuss the interaction of radiation and engineered NPs that lead to the generation of reactive oxygen species, as illustrated in **Figure** **2**. Section 4 centers on the interaction of NPs with radiation and related physical and chemical effects. The discussion is developed through the lens of catalytic processes at solid surfaces. Drawing on analogies between photo‐ and radio‐catalysis, we suggest future developments of the X‐PDT field that build on selected advances in the areas of clean energy, water splitting, and environmental remediation. In Section 5 we focus on nanomaterials in which the coatings contain clinically used PSs, which offer additional opportunities for maximizing the efficacy, by utilizing the transduction of ionizing radiation into visible light or the Cherenkov light generated in tissue by the passage of ionizing radiation. Section 6 discusses example particle designs that include PSs and their interaction with radiation. Focusing on future clinical translation, we discuss biocompatible nanocarriers (liposomes and poly(lactic‐*co*‐glycolic acid) (PLGA) NPs) as well as mesoporous silica and point to novel opportunities related to aggregation‐induced emission. Section 7 clarifies the issue of somewhat fluid terminology used in the field. Finally, in Sections 8 and 9 we draw a roadmap of future development in this field and discuss the opportunities and challenges in the translation of X‐PDT into clinical oncological practice.

## 2 Interaction of High Energy Electromagnetic Radiation with Materials: Physical and Chemical Mechanisms

### 2.1 Primary Molecular Damage Induced by Ionizing Radiation

The term “ionizing radiation” refers to electron, proton, and neutron beams or electromagnetic radiation with high enough energy to ionize atoms and molecules. High energy electromagnetic radiation is conventionally referred to as X‐rays if they have energy higher than ≈12–120 eV (100–10 nm wavelength), and we remind that visible light is also a form of electromagnetic radiation but with energies in the range of 1.8 to 4.1 eV (700 to 300 nm wavelength). If the electromagnetic rays are generated via a nuclear decay, they are typically referred to as gamma (*γ*) rays.^[^ ^54^ ^]^ Clinical radiotherapy uses either external X‐ray beams, nowadays mostly high‐energy (up to about 20 MeV) from linear accelerators, or internal radioactive implants (brachytherapy), depending upon the location and type of tumor. Interactions of these X‐rays with atoms in the tissue^[^ ^55^, ^56^ ^]^ result in a cascade of secondary photoelectrons and Auger electrons. These disrupt atomic and molecular structures along the radiation track, eventually dissipating their energy and leaving in their wake a pool of reactive and potentially cytotoxic molecular species, including (thermalized) hydrated electrons, free radicals, and various excited‐state ions and molecules. X‐rays have a low LET of 0.2–0.3 keV µm^−1^, compared, for example, with alpha particles which have high LET ≈140 keV µm^−1^.^[^ ^57^ ^]^ The penetration depth of ionizing radiation is inversely related to its LET,^[^ ^58^ ^]^ as and the value of LET plays a significant role in the induction of cellular damage. High LET radiation causes more extensive cellular damage, but it does not penetrate as deeply into the body as low LET radiation that is typically used to treat deep‐seated tumors. The radiation dose, D, in tissue is expressed in Grays (Gy), where 1 Gy = 1 J of energy absorbed per kg of tissue mass.^[^ ^59^ ^]^ In order to account for different biological effects of ionizing radiation with varying LET, the concept of “equivalent dose” is also used. The equivalent dose is measured in units of Sieverts (Sv), and it is given by the product of D and a dimensionless quality factor, *Q*, that depends on the LET.

Many of these constituents are transient, with sub‐µs lifetimes.^[^ ^57^ ^]^ Since water is the main constituent of cells and tissues by mass (>80%), the primary effect of X‐rays is radiolysis of water to produce radiolytic products.^[^ ^57^, ^60^ ^]^ The balance of radiolytic products ($e_{\mathrm{aq}}^{-}$, H^•^, HO^•^, ${\mathrm{HO}}_{2}^{•}$, OH^−^, H_3_O^+^, H_2_, H_2_O_2_) depends on the linear energy transfer (LET) of the radiation, i.e., energy (E) deposited per unit distance (*x*) along the ionizationtrack^[^ ^56^ ^]^

(1) $$\begin{array}{c} \mathrm{LET}=-\frac{\mathrm{dE}}{\mathrm{dx}} \end{array}$$

X‐rays have a low LET of 0.2–0.3 keV µm^−1^, compared, for example, with alpha particles which have high LET ≈140 keV µm^−1^.^[^ ^57^ ^]^ The penetration depth of ionizing radiation is inversely related to its LET,^[^ ^58^ ^]^ as and the value of LET plays a significant role in the induction of cellular damage. High LET radiation causes more extensive cellular damage, but it does not penetrate as deeply into the body as low LET radiation that is typically used to treat deep‐seated tumors. The radiation dose, D, in tissue is expressed in Grays (Gy), where 1 Gy = 1 J of energy absorbed per kg of tissue mass.^[^ ^59^ ^]^ In order to account for different biological effects of ionizing radiation with varying LET, the concept of “equivalent dose” is also used. The equivalent dose is measured in units of Sieverts (Sv), and it is given by the product of D and a dimensionless quality factor, *Q*, that depends on the LET.

### 2.2 Light Generation by Radiation: Cherenkov Effect

The passage of high‐energy secondary electrons generated by the MeV range radiation through a dielectric medium such as tissue can also generate Cherenkov light (CL).^[^ ^61^, ^62^ ^]^ This requires X‐rays (or gamma rays) of energy higher than the Cerenkov threshold (*E*_T_), whose value in tissue is 219 keV.^[^ ^63^, ^64^, ^65^ ^]^ In vacuum CL has a broad spectrum that varies as a function of wavelength, *λ*, as *λ* ^−2^.^[^ ^66^ ^]^ Due to light absorption and scattering in tissue and its components (water, hemoglobin, lipids), the effective CL spectrum in tissue has a complex shape, with a maximum around 650 nm.^[^ ^67^, ^68^, ^69^, ^70^ ^]^

The estimated photon yield of the Cherenkov process from clinical radionuclides is rather low, ≈1–50 photons per decay, corresponding to ≈12 000 photons per Bq for ^18^F and ≈199 000 for ^68^Ga.^[^ ^66^ ^]^ The Cherenkov photon yield from 5 to 20 MeV X‐ray photon beams is 60–100 photons per deposited MeV of energy.^[^ ^66^ ^]^ The latter value can be related to the radiation dose where for 1 Gy radiation dose, ≈5 × 10^11^ Cherenkov photons are generated per cm^3^ of tissue. Correspondingly, the CL intensity reported in the literature is low (≈0.01–1 nW cm^−2^ per MBq g^−1^ for radionuclides, and 1–100 µW cm^−2^ per Gy s^−1^ for external radiotherapy beams).^[^ ^70^ ^]^ Despite this weakness of the CL, several authors have attributed an improved anticancer effect of their nanomaterials combined with radioisotopes to a Cherenkov process.^[^ ^66^, ^71^, ^72^ ^]^

### 2.3 Primary Chemically Active Species Generated by Ionizing Radiation

**Figure 3:**
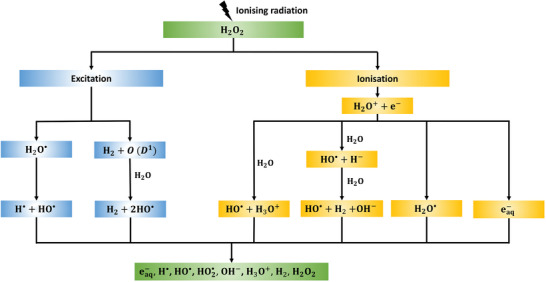
Main molecular events during radiolysis of water and key radiolytic products ($e_{\mathrm{aq}}^{-},\;H^{•},\;HO^{•},{\;\mathrm{HO}}_{2}^{•},\;OH^{-},\;H_{3}O^{+},\;H_{2},\;H_{2}O_{2}$). Adapted with permission under the Creative Commons Attribution License.^[^ ^57^ ^]^ Copyright 2011, MDPI.

The secondary electrons produced by ionizing radiation are highly reactive and generate multiple radiolytic products, either directly or indirectly.^[^ ^57^, ^73^ ^]^ The molecular events during the radiolysis of water are described in **Figure** **3**. The main products of low‐LET radiation in water are $e_{\mathrm{aq}}^{-}$ (hydrated electrons), HO^•^ (hydroxyl radicals), H^•^ (hydrogen radicals), H_2_ (radiolytic hydrogen), H_2_O_2_ (hydrogen peroxide), and ${\mathrm{HO}}_{2}^{•}$ (hydroperoxyl radical) (see **Table** **1** for radiolytic yields^[^ ^57^ ^]^).

**Table 1:** Radiolytic yield, half‐life and approximate migration distance of main radiolysis products in water. The radiolytic yield (*G* value) represents the number of molecules created or destroyed per 100 eV of energy deposited in the system and half‐life is the time required for a quantity to reduce to half of its initial value

Radiolytic product	Migration distance [m]	Half‐life [s]	Radiolytic yield [µmol J^−1^]
HO^•^	4 × 10^−9^–6 × 10^−9[^ ^74^ ^]^	10^−9[^ ^79^ ^]^	0.28^[^ ^57^ ^]^
H^•^	9 × 10^−9^–4 × 10^−7[^ ^74^ ^]^	10^−9[^ ^74^ ^]^	0.06^[^ ^57^ ^]^
H_2_O_2_	1 × 10^−6[^ ^75^ ^]^	10^−3[^ ^76^ ^]^	0.063^[^ ^57^ ^]^
$e_{\mathrm{aq}}^{-}$	1 × 10^−6^–2 × 10^−6[^ ^74^ ^]^	2 × 10^−5^–8 × 10^−4[^ ^77^ ^]^	0.28^[^ ^57^ ^]^

H_2_O_2_ is the only stable product of radiolysis, while the other products are transient; e.g., the half‐life of HO^•^is ≈10^−9^ s.^[^ ^78^, ^79^ ^]^ H_2_ is not relevant for radiotherapy, as it escapes from the aqueous solution, whereas $e_{\mathrm{aq}}^{-}$ and H^•^ convert oxygen to $O_{2}^{-}$ (superoxide) or ${\mathrm{HO}}_{2}^{•}$ radicals, the latter being present in only negligible amounts for low‐LET radiation.^[^ ^57^ ^]^ Consequently, HO^•^, H_2_O_2_, and oxygen level‐dependent $O_{2}^{-}\;$are the main ROS formed in cells during low‐LET radiolysis of water,^[^ ^57^, ^60^, ^80^ ^]^ while $e_{\mathrm{aq}}^{-}$ and H^•^ are also transiently present and limited by the concentration of available oxygen.

Representative one‐electron redox reactions generating some of these products, and the associated reduction potentials *ε*′, are given by^[^ ^81^ ^]^

(2) $$\begin{array}{c} O_{2}+e^{-}\to O_{2}^{-},\;\varepsilon^{\prime }=-0.33\;V \end{array}$$

(3) $$\begin{array}{c} O_{2}^{-}+2H^{+}+e^{-}\to H_{2}O_{2},\;\varepsilon^{\prime }=+0.89\;V \end{array}$$

(4) $$\begin{array}{c} H_{2}O_{2}+H^{+}+e^{-}\to H_{2}O+HO^{•},\;\varepsilon^{\prime }=+0.38\;V \end{array}$$

The cited values are relative to the potential of a neutral hydrogen electrode and at pH = 7.

The key radiolytic products in water are the same as the main physiological ROS generated in cells,^[^ ^78^ ^]^ but their absolute and relative concentrations differ. In particular, in low‐LET irradiation of cells, HO^•^ is the most abundant ROS, while $O_{2}^{-}$ and H_2_O_2_ are the key species produced by endogenous processes in cells through multiple mechanisms.^[^ ^60^ ^]^ There are further differences in the dynamics between ROS that are physiologically generated and those created by water radiolysis, in particular ROS bursts and clustering of radiation damage is found in cells exposed to X‐ray radiation, compared with a more homogeneous distribution of endogenous physiologically generated ROS.^[^ ^60^ ^]^

On average, about 30 eV of energy is required to generate a single ROS radical by radiolysis of water.^[^ ^82^ ^]^ Hence, a short (<1 µs) burst of about 200 ROS is created by a single low LET X‐ray photon depositing ≈6 keV µm^−1^ in a 20 µm long track within a cell.^[^ ^82^ ^]^ By contrast, the ROS generation rate from cellular metabolism is some three orders‐of‐magnitude lower, at ≈10^5^ ROS per second per cell.^[^ ^82^ ^]^

The primary products of water radiolysis are readily converted in cells into further reactive species. For example, superoxide can react with molecular hydrogen to generate hydrogen peroxide^[^ ^83^ ^]^

(5) $$\begin{array}{c} 2O_{2}^{-}+2H\to H_{2}O_{2} \end{array}$$

The primary ROS, especially HO^•^, then react further to create organic radicals (R•) and, by a subsequent rapid reaction with O_2,_ strongly oxidizing peroxyl radicals (${\mathrm{RO}}_{2}^{•}$). These reactive intermediates then interact with available H^•^ to form hydroperoxides (ROOH).^[^ ^60^ ^]^

Hydrated electrons produced in water radiolysis are very powerful reductants capable, for example, of reducing metal ions that may then react further with oxygen, giving rise to superoxide

(6) $$\begin{array}{c} M{e_{\mathrm{red}}}^{n+}+O_{2}↔M{e_{\mathrm{ox}}}^{\left(n+1\right)+}+O_{2}^{-} \end{array}$$

Radiolytically generated hydrogen peroxide, H_2_O_2_, in the presence of redox‐active metals such as iron, copper, manganese or zinc ions present in cells that serve as catalysts may form free hydroxyl radicals through the Fenton, Fenton‐like or the Haber–Weiss cycle reactions.^[^ ^84^ ^]^ The Fenton reaction generates hydroxyl radicals and increases the charge state of metal ions according to the following reaction

(7) $$\begin{array}{c} Fe^{2+}+H_{2}O_{2}\to Fe^{3+}+HO^{•}+OH^{-} \end{array}$$

Similarly, Fenton‐like reactions increase the charge state of copper to produce hydroxyl radicals

(8) $$\begin{array}{c} Cu^{+}+H_{2}O_{2}\to Cu^{2+}+HO^{•}+OH^{-} \end{array}$$

The Haber–Weiss cycle reaction generates hydroxyl radicals from H_2_O_2_ and superoxide anions, while the metal ions are cycled between their charged states

(9) $$\begin{array}{c} O_{2}^{-}+\;H_{2}O_{2}\to \;•\mathrm{OH}+OH^{-}+O_{2} \end{array}$$

Furthermore, radiolytically generated ROS may react with nitric oxide radicals (NO^•^) that are endogenously abundant in cells.^[^ ^78^ ^]^ These radicals have half‐lives of a few seconds in an normoxic aqueous environment and >15 s in a hypoxic environment.^[^ ^78^ ^]^ NO^•^ combines, for example, with a superoxide anion to form peroxynitrite anion (ONOO^−^)

(10) $$\begin{array}{c} NO^{•}+O_{2}^{-}\to \mathrm{ONO}O^{-} \end{array}$$

This is a potent oxidant that can further bind with transition metal ions such as iron and copper.^[^ ^78^ ^]^ This and other reactive nitrogen species (RNS) elicit further damage in cells, such as lipid peroxidation and damage to proteins and DNA.^[^ ^60^, ^85^ ^]^

The products of radiolysis may also enter further biochemical enzymatic reactions in cells enabled by cellular mechanisms that maintain homeostasis of endogenous ROS species. For example, the superoxide in cells is converted by superoxide dismutase into hydrogen peroxide and singlet oxygen (^1^O_2_).^[^ ^86^ ^]^

The half‐lives of some ROS/RNS generated by light and/or radiation, in particular for hydroxyl radicals and ^1^O_2_ are very short (<1 µs), so that they act very locally, typically within tens of nm from the generation site.^[^ ^87^ ^]^ The much lower reactivity of H_2_O_2_ and $O_{2}^{-}$ and longer half‐lives allow them to diffuse further from the site of origin to other cellular compartments.

The short life of the ROS/RNS makes their detection challenging. Direct approaches, such as electron paramagnetic (EPR) spectroscopy and near‐infrared luminescence spectroscopy are limited by the short half‐lives so that observation is possible only on a sub‐ms time scale,^[^ ^88^ ^]^ and typical low concentrations. Near‐infrared spectroscopy detection is based on the luminescent emission of ^1^O_2_ at 1270 nm, which is very weak (due to competing de‐excitation pathways of biomolecular interactions), leading to low sensitivity.^[^ ^88^, ^89^, ^90^ ^]^ Alternative but indirect methods use high‐sensitivity probes^[^ ^91^, ^92^ ^]^ suitable for fluorimetry or fluorescence imaging;^[^ ^93^, ^94^ ^]^ these overcome the short lifetimes and low concentration limitations.^[^ ^95^ ^]^ Commonly used probes include 1,3‐diphenyl isobenzofuran (DPBF), 9‐[2(3‐carboxy‐9,10‐dimethyl)anthryl]‐6‐hydroxy‐3H‐xanthen‐3‐one(DMAX),9‐[2‐(3‐carboxy9,10‐diphenyl)anthryl]‐6‐hydroxy‐3H‐xanthen‐3‐one(DPAX), singlet oxygen sensor green (SOSG: dichlorodihydrofluorescein), and hydroethidine, as well as dihydrorhodamine and chemiluminescent methods^[^ ^96^, ^97^ ^]^ are also used. •OH radicals can be quantified using coumarin scavenging, where the resulting 7‐hydroxycoumarin fluorescence is measured.^[^ ^98^, ^99^ ^]^ Hydrated electrons can be been quantified by determining the •OH generation measured under N_2_O atmosphere and comparing it to the value obtained under N_2_.^[^ ^99^ ^]^ The main advantages of using chemical probes compared to direct methods are their strong signals and the corresponding ease of fluorescence detection in the visible spectral range. Drawbacks include the need to use an additional exogenous (and potentially toxic) materials, the possibility of generating additional reactive photoproducts, and the confounding effects of the microenvironment.^[^ ^99^ ^]^ Nosaka and Nosaka^[^ ^100^ ^]^ provide an extensive summary of the available ROS detection methods.

## 3 Interaction of Radiation with Cells and Tissues: Biological Effects

### 3.1 Reactions of Radiolysis Products with Biomolecules in Cells

Low energy electrons such as $e_{\mathrm{aq}}^{-}$, free radicals with unpaired electrons, as well as other secondary reactive oxygen and nitrogen species, show variable reactivity toward critical components of cells.^[^ ^60^ ^]^ For example, superoxide is a poor oxidant with low reactivity, in contrast to the hydroxyl radical which is highly reactive due to its unpaired spin.^[^ ^101^ ^]^ These different species may cause lipid peroxidation or protein denaturation.^[^ ^78^, ^101^, ^102^ ^]^ Their interactions with nucleic acids can induce structural DNA damage (nuclear and mitochondrial), such as single or double strand breaks (SSB, DSB), as well as crosslinking and telomere dysfunction, particularly when damage clusters are formed. The DNA damage mediated by low energy electrons is initiated by electron excitation in DNA molecules which leave behind positively charged holes. These migrate along the DNA molecule, accumulating mainly in guanine, which has the lowest ionization potential among all nucleobases, and the ensuing base damage, assisted by endonuclease and glycosylase, produces strand breaks.^[^ ^103^ ^]^ Two thirds of the total DNA damage is due to low energy electrons and the remainder is caused by the interaction with ROS.^[^ ^104^ ^]^

The reactivity and/or generation of free radicals may be enhanced by exogenous drugs such as chemotherapy agents and radiosensitizers.^[^ ^16^, ^54^, ^105^, ^106^, ^107^ ^]^ For example, the incorporation into DNA of platinum complexes such as cisplatin, oxaliplatin or carboplatin can increase the reactivity of low‐energy electrons by a factor of 2–4, which enhances the radiotherapeutic efficacy. Anticancer drugs such as doxorubicin, epirubicin, and daunorubicin also act as ROS‐generating agents in their own right.^[^ ^84^ ^]^

Given the significance of cellular DNA damage, efforts have been expended to optimize it, for example, by nuclear radiosensitization^[^ ^108^ ^]^ or by targeting NPs directly to the nucleus^[^ ^109^ ^]^ using cell‐penetrating peptides (CPPs), such as transactivating transcriptional activator from human immunodeficiency virus 1 (TAT), nuclear localization sequence or arginylglycylaspartic acid (RGD) peptides.^[^ ^110^, ^111^, ^112^ ^]^ Nanocarriers with such targeting moieties can deliver biologically active cargoes (drugs, genes, antibodies, imaging agents, etc.) and even other small NPs to the nucleus.^[^ ^113^ ^]^ For example, nuclear targeting of gold NPs with TAT peptide resulted in a radiation sensitization enhancement ratio (SER) of 2.3.^[^ ^114^ ^]^ Alternatively, 7‐Ethyl‐10‐hydroxy‐camptothecin (SN‐38) has been delivered to cells using nuclear‐targeted mesoporous TiO_2_ NPs, achieving G2 cell cycle arrest in the most radiosensitive G2 phase.^[^ ^115^ ^]^

In addition to water, molecular oxygen is a major participant in many reactions generating ROS from radiolytic products. Its concentration in cells and tissues is critical for the relative abundance of the various ROS generated by ionizing radiation. Radiation‐induced ROS generation in vivo is limited by tumor hypoxia caused by inadequate blood supply. This has been addressed clinically by approaches such as hyperbaric oxygen, blood substitutes that carry oxygen, or hypoxic radiosensitizer drugs such as misonidazole, metronidazole, and tirapazamine that selectively kill hypoxic cells.^[^ ^104^, ^116^ ^]^ NPs have also been explored preclinically to improve radiosensitization as well as to address tumor hypoxia, including hafnium oxide NPs (NBTXR3: Nanobiotix, USA, currently in clinical trials) or gold and manganese dioxide core–shell NPs with a polyethylene glycol (PEG) coating.^[^ ^117^ ^]^ For example, administering different formulation of MnO_2_ NPs (hydrophilic terpolymer–protein–MnO_2_ and hydrophobic polymer–lipid–MnO_2_) in highly hypoxic murine or human xenograft breast tumor models increased the radiotherapy efficacy, reducing tumor growth and VGRF expression.^[^ ^118^ ^]^ In this work 40% of tumor‐bearing mice became tumor free after a single treatment with a 2.5‐fold lower radiation dose than that required to achieve same outcome without NPs.

### 3.2 Cellular Antioxidant Response to ROS Generated by Radiation

Cells are not in thermodynamic equilibrium with the environment due to internal energy generation, but rather support a nonequilibrium steady redox state.^[^ ^119^ ^]^ The overall cellular redox state reflects the state of multiple individual redox couples such as NAD^+^/NADH, some of which are linked.^[^ ^120^, ^121^ ^]^ Quantitatively, the effective reduction potential (E^eff^) of a redox system such as a cell is defined by

(11) $$\begin{array}{c} E^{\mathrm{eff}\;}=\;\sum_{i\;=\;1}^{n}a_{i}\;E_{i} \end{array}$$

where the index, *i* = 1, …*n*, denotes different redox couples and

(12) $$\begin{array}{c} \;a_{i}=\frac{C_{i}Z_{i}}{\sum_{j\;=\;1}^{n}C_{j}Z_{j}} \end{array}$$

is the specific charge transferred in a given reaction, *E_i_* is the reduction potential, *c_i_* is the molar concentration of reduced species, and *z_i_* is the number of electrons which transfer to the oxidized species of a given couple in one redox reaction.^[^ ^120^ ^]^ Under normal conditions the redox state is tightly regulated, with intracellular ROS production is balanced by the antioxidant system to maintain cellular homeostasis.

Antioxidants are reducing agents whose role is to neutralize the ROS and free radicals generated as a result of respiration, metabolism, cell signaling, and other processes and environmental exposure. These antioxidants include tocopherols and ascorbic acid, carotenoids, and other flavonoids, and peptides such as glutathione, the antioxidant enzymes include superoxide dismutase, catalase and glutathione peroxidase, the thioredoxin system, and free amino acids.^[^ ^84^, ^86^ ^]^ Glutathione is the most abundant cellular antioxidant, with concentrations of 1 × 10^−3^–11 × 10^−3^ m in the cytosol, 3 × 10^−3^–15 × 10^−3^ m in the nucleus and 5 × 10^−3^ –11 × 10^−3^ m in the mitochondria.^[^ ^78^ ^]^ Due to its high abundance, the reduction potential of the glutathione/glutathione disulphide couple can be considered as a surrogate for the overall cellular redox state.^[^ ^119^, ^121^ ^]^ Disturbing the cellular redox state by affecting the balance between ROS production and elimination leads to oxidative stress. The exogenous formation of free radicals in cells increases oxidative stress^[^ ^60^, ^121^, ^122^ ^]^ and more than 60% of the cellular damage caused by X‐rays is due to this effect.^[^ ^123^ ^]^

ROS are endogenously generated in cells at multiple sites, including mitochondria, endoplasmic reticulum, and lysosomes/peroxisomes in the course of metabolism and autophagy, as well as in response to cytokines, xenobiotics, and microbial invasion.^[^ ^124^ ^]^ The molecules and processes mobilized by cells to respond to ROS/RNS generated by radiation are the same as those regulating the cellular antioxidant system,^[^ ^125^ ^]^ for example, upregulation of cyclooxygenases, nitric oxide synthases, lipoxygenases, and nicotinamide adenine dinucleotide phosphate oxidase (NADPH oxidase), and the Nrf2 antioxidant response.^[^ ^125^, ^126^ ^]^ This upregulation activates critical cytoprotective defences. These regulatory mechanisms offer adequate protection at low ROS doses/dose rates but become inefficient at higher doses/dose rates.^[^ ^86^ ^]^

It is also noteworthy that cells exhibit natural variations in free‐radical responses, related to cell cycle or circadian cycles. Significant differences in radiation sensitivity are observed in various phases of the cell cycle, being most resistant in late S phase and most sensitive in mitotic G2/M phase: G2/M > G1 > early S> late S.^[^ ^127^ ^]^ Additionally, the expression levels of many ROS‐responsive genes or antioxidant enzymes are regulated by the clock genes.^[^ ^128^ ^]^

Weakening the antioxidant response and/or blocking antioxidant synthesis can accelerate cell death, in a way that is cancer selective. This can be achieved with antioxidative enzyme inhibitors, such as l‐buthionine sulfoximine, a drug which inhibits glutamate‐cysteine ligase that is the rate‐limiting enzyme in glutathione synthesis. Other drugs, including b‐phenylethyl isothiocyanate, zinc protoporphyrin, dimethylfumarate, and diethylmaleate, deplete biologically‐active GSH and amplify ROS‐induced oxidative stress.^[^ ^104^ ^]^ By using this approach, ROS levels can be modulated by tailored NP interventions. Recent work has shown, for example, that GdVO_4_:Eu_3_^+^ NPs increase ROS levels in X‐ray irradiated cells, whereas CeO_2_ NPs inhibit ROS generation under the same conditions.^[^ ^129^ ^]^ Glutathione‐depleting NPs (histidine‐coated gold nanoclusters) have been shown to suppress antioxidant cell responses to ROS,^[^ ^130^ ^]^ offering an alternative way of amplifying oxidative stress. These NPs were able to arrest a significant proportion of cells at the radiosensitive G2/M phase, while in vivo studies confirmed tumor inhibition and fast renal clearance of the NPs. Thus, antioxidant response suppression provides a new NP‐based strategy to enhance cancer radiotherapy.

### 3.3 Cellular Repair of Radiation‐Induced DNA Damage

Cells engage dedicated mechanisms to restore the integrity of damaged DNA. The SSBs are recognized by enzymes from the poly (ADP‐ribose) polymerase (PARP) family, such as PARP1, and rectified by the base‐excision repair machinery.^[^ ^82^ ^]^ The DNA damage response is additionally orchestrated by phosphatidylinositol 3‐kinase (PI3K), mitogen‐activated protein kinase (MAPK), and SIRT pathways. Furthermore, DNA damage signals activate cell cycle checkpoints and arrest cells in the G2/M phase, to provide time for DNA repair systems to work.^[^ ^131^ ^]^ The ATM and ATR kinase signaling cascades are also initiated.^[^ ^104^ ^]^ The DNA repair can be hindered, for example, by using PARP inhibitors^[^ ^132^, ^133^ ^]^ or by knocking down PARP expression with siRNA, which may be delivered by using NPs.^[^ ^134^ ^]^ Such blocking of DNA repair enhances the effectiveness of ROS generated by limited radiation doses.

### 3.4 Cellular Pathways Affected by Radiation

**Figure 4:**
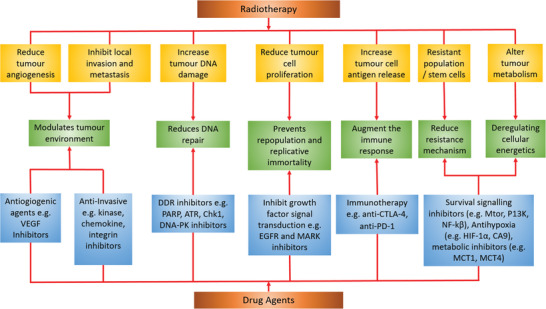
Combining radiation with agents that target the specific cellular pathways offers new tumor treatment strategies. ATR, ataxia telangiectasia and Rad3‐related protein; CA9, carbonic anhydrase 9; Chk1, checkpoint kinase 1; CTLA‐4, cytotoxic T‐lymphocyte‐associated protein 4; DDR, DNA damage response; DNA‐PK, DNA‐dependent protein kinase; HIF‐1‐*α*, hypoxia‐inducible factor 1‐alpha; MCT 1, monocarboxylate transporter 1; MCT 4, monocarboxylate transporter 4; mTOR, mechanistic target of rapamycin; PARP, poly(ADP‐ribose) polymerase; PD‐1, programmed cell death protein 1; PI3K, phosphoinositide 3‐kinase; NF‐*κ*B, nuclear factor‐kappa‐B. Adapted with permission under the Creative Commons Attribution License.^[^ ^164^ ^]^ Copyright 2016, Springer Nature.

The key mechanisms underlying cellular response to ionizing radiation have been the focus of many studies and multiple pathways have been found to contribute.^[^ ^135^, ^136^, ^137^ ^]^ These involve DNA repair machinery, controlling cell cycle checkpoints, cellular senescence, autophagy, apoptosis, cell metabolism, etc., each linked to multiple cellular pathways. For example, DNA damage activates the G1/S and G2/M cell cycle checkpoints to allow more time for DNA repair,^[^ ^133^ ^]^ so that tumors can be radiosensitized by drugs that block the activation of the G2/M checkpoint. Radiation affects the HIF‐1 pathway, enhancing glycolysis and also the pentose phosphate pathway, which increases the production of antioxidants that buffer ROS.^[^ ^104^ ^]^ Radiation‐induced ROS also affect the glucose transporter 1 (GLUT1) mechanism essential for glucose metabolism. The AKT/mTOR/STAT3 signaling pathway upregulated by radiation activates several epithelial‐mesenchymal transition transcription factors, including SNAI1, HIF‐1, ZEB1, and STAT3, thereby promoting cancer cell metastasis. The proteins ATM, ATR, and downstream kinases, as well as the proapoptotic proteins BAX and p53 may also be upregulated. The Ras/MAPK/ERK and Ras/PI3K/AKT signaling pathways involved in regulation of cell proliferation, survival, differentiation, and angiogenesis may also be affected by radiation.^[^ ^133^, ^138^ ^]^ Cell adhesion molecules may be upregulated by radiation which promotes cancer invasion. The expression of tumor‐suppressing miRNAs, such as miR‐29c and miR‐22, may also be altered.^[^ ^104^, ^138^ ^]^ It is worth mentioning other in vivo responses to radiation, including alterations in tumor microenvironment such as extracellular matrix alterations, angiogenesis a well as the immune response (**Figure** **4**). Radiation‐induced DNA and membrane damage, as well as cytoplasmic ROS, activate many transcription factors and signaling pathways (including mTOR, IGF, and CXCL8) that modulate the immunophenotype and immunogenicity of tumor cells.^[^ ^139^ ^]^ Importantly, radiation upregulates the expression of immune‐checkpoint ligands, including PD‐L1, on the surface of tumor cells and on immune cells in the tumor microenvironment. This, in turn enhances the density of immune‐cell infiltrates allowing the immune system to fight the tumor.^[^ ^139^ ^]^

### 3.5 Other Cellular Responses to Radiation: Resistance Mechanisms

Radiotherapy aims to prevent cell proliferation by inducing cellular responses such as necrosis, apoptosis or senescence.^[^ ^140^, ^141^, ^142^, ^143^ ^]^ In apoptosis and necrosis, the damaged cells die, while senescence arrests the cell cycle. If ROS exposure induces a sufficient level of oxidative stress then no controlled pathway to cell death can be followed, resulting in necrosis, characterized by rupture of the cytoplasmic membrane, explosive release of cellular contents, and massive inflammatory response in vivo. These responses can be induced intentionally, for example, by using membrane‐targeted NPs that generate ^1^O_2_ induce necrosis by destroying cell membranes.^[^ ^144^ ^]^ Excessive levels of ROS have been linked to apoptotic cell death, including death mediated by mitochondrial, cell‐death receptor and/or endoplasmic reticulum pathways.^[^ ^145^ ^]^ Cells can also undergo autophagy, a process by which they reabsorb their own components, leading to self‐destruction or survival, depending on the conditions.^[^ ^133^ ^]^ Autophagy may be triggered at more modest levels of ROS than required for necrosis. At low‐enough levels of radiation‐generated ROS, cells can mount transient responses, including DNA repair and antioxidant generation, resulting in proliferative survival.

As radiation is reliant on the generation of ROS, a hypoxic environment where unbound oxygen is scarce represents one of the major components of resistance by which radiotherapy becomes ineffective. In hypoxia, the hypoxia‐inducible transcription factors (HIF), key regulators of the hypoxic response become overexpressed via HIF prolyl hydroxylases (PHDs).^[^ ^146^, ^147^ ^]^ These enzymes rely on oxygen for HIF degradation, thus, during hypoxia, PHDs become inactive, and HIF may accumulate. Alternatively, HIF activation is also mediated through signaling mechanisms including the unfolded protein response and mTOR signaling.^[^ ^147^ ^]^ These independent pathways which are integrated with each other and within HIF activation ultimately increase HIF signaling.^[^ ^148^ ^]^ HIF modulates genes which aid in adapting the cell to hypoxic conditions by reducing oxygen consumption via shifting energy metabolism to the glycolytic pathway which requires less oxygen than the alternative oxidative phosphorylation pathway.^[^ ^149^ ^]^ Additionally, HIF stimulates angiogenesis which increases vascular flow to hypoxic tumor areas.^[^ ^149^ ^]^ The increase in glycolytic metabolism leads to the production of lactate, which further acidifies the extracellular environment.^[^ ^150^ ^]^ The combination of acidification along with altered angiogenesis created an environment in which low levels of oxygen are available for radiolytic ROS generation environment.^[^ ^150^ ^]^

### 3.6 Pharmacological Radiosensitizers

Pharmacological methods of radiosensitization by interfering with cellular processes during and post radiation have been well established, and many clinical radiosensitizers have been developed. Most commonly used clinical radiosensitizers include cisplatin, 5‐fluorouracil (5‐FU), gemcitabine, and taxanes.^[^ ^12^, ^151^, ^152^ ^]^ Some radiosensitizers (e.g., nitroimidazole/intercalator conjugates, nitroquinoline intercalators) act by intercalating with the DNA and destabilizing its structure so that it is more easily damaged by X‐rays.^[^ ^12^ ^]^ Others directly interfere with specific cellular pathways, for example, enhanced apoptosis can be accomplished by increasing the activity of specific (proapoptotic) genes or by decreasing their activity using inhibitors. Alternatively, similar interventions may be applied upstream or downstream of the relevant cellular pathways. As an example, histone deacetylases (HDACs) are required for DNA repair following radiation. Correspondingly, the application of HDAC inhibitors such as Vorinostat increase DNA radiation damage.^[^ ^153^ ^]^ **Table** **2** provides a list of clinically approved radiosensitizers and their mechanisms‐of action‐including their target pathways and also their radiation sensitization factor. This factor also variably called “an SER” or “radiation dose enhancement (DEF)” determines the ratio of doses without and with radiosensitizers which have the same biological effectiveness. This term is conceptually simple but difficult to determine due to scarcity of coherent and systematic experimental approaches to fully evaluate the radiobiological effectiveness of different agents^[^ ^154^ ^]^ There seems to be consensus that for most of the clinical sensitizers the SERs/DEFs are in the range of 1.2 to 1.3 (a 20% to 30% increase in the effective dose to the tumor^[^ ^155^ ^]^ (noting that it is cancer and tissue‐dependent). Higher values are also reported in the literature for less frequently used clinical preclinical radiosensitizers, as seen in Table 2, e.g., 1.79 (79% increase) was reported.^[^ ^156^ ^]^ Various (preclinical) types of gold NPs reviewed by Her et al.^[^ ^24^ ^]^ are reported to have SERs/DEFs in the range of 1–3 (up to 200% increase).

**Table 2:** Examples of clinically approved radiosensitizers

Drug	Mechanism of action	Target pathway	Radiation dose enhance‐ment factor	Conditions	Ref.
CC2	Inhibits EGFR receptor to inhibit VEGF production and tumor angiogenesis	EGFR	1.59–3.62	Normal	^[^ ^157^ ^]^
Celotoxib	Enhanced radiation‐induced G2‐M arrest	COX‐2	1.2–1.9	Normal	^[^ ^158^ ^]^
Gemcitabine	Causes S‐phase arrest	Chk1	1.3–1.8	Normal	^[^ ^159^, ^160^ ^]^
Olaparib (AZD2281)	Delays DNA double strand break repair	PARP1	1.4–2.5	Normal	^[^ ^161^ ^]^
Pentoxifylline	Inhibits G2/M block reducing time between DNA repair and mitosis	PDE	1.3–6.0	Hypoxic	^[^ ^162^ ^]^
Perifosine	Enhances radiation‐induced apoptosis	Akt	1.3	Normal	^[^ ^163^ ^]^
Vorinostat	Inhibits NHEJ and HR DNA repair system and abrogates EGFR and NF‐*κ*B signaling	HDAC	1.4–1.6	Normal	^[^ ^153^ ^]^

The radioresistance pathways and mechanisms have been well investigated and a large number of approved or candidate drugs exist that interfere with key components of these pathways (Figure 4, see also ref. ^[^ ^73^ ^]^). We listed in Table 2 some example radiosensitizer drugs as many of them may be well‐suited to combining with the nanomaterial optimization strategies discussed below. Thereby, the cellular ROS response can be manipulated to increase vulnerability of cancer cells, maximize tumor ablation and increase the effectiveness of the radiation dose.

## 4 Interaction of Radiation with Nanoparticles: Physical and Chemical Effects

We now summarize the interaction mechanisms of radiation with NPs that may be utilized to maximize the ROS generation rates.

### 4.1 Dose Partitioning

Dose partitioning is the primary effect that may occur in and around NPs located within tissue when they interact with ionizing radiation. This effect is driven by the difference in atomic mass in NPs, compared with the average atomic number in cells/tissues of ≈3–7, and it is most pronounced for high *Z* (atomic number) NPs. In dose partitioning, an increased fraction of the X‐ray photon energy is deposited close to NPs such as gold (*Z* = 79), as a result of increased photoelectric interactions,^[^ ^165^ ^]^ thereby enhancing the local radiation dose.^[^ ^166^, ^167^, ^168^, ^169^, ^170^ ^]^ Monte Carlo simulations have established that combining a typical radiation treatment with Au NPs for ≈100 keV X‐rays and 0.2–0.6 MeV *γ*‐rays from ^192^Ir results in a >10% dose enhancement for achievable gold concentration of 7 mg Au per gram in tumors,^[^ ^165^ ^]^ although the dose enhancement at typical radiotherapy energies of > ≈4–6 MeV is not highly pronounced. Radiation dose enhancement has been reported also for other metal‐containing NPs, including platinum,^[^ ^171^ ^]^ iridium,^[^ ^172^ ^]^ selenium^[^ ^173^ ^]^ Fe–Pt clusters,^[^ ^174^ ^]^ superparamagnetic iron oxide,^[^ ^175^ ^]^ as well as gadolinium MRI‐contrast agents,^[^ ^176^ ^]^ as reviewed by Liu et al.^[^ ^177^ ^]^ In addition to the effect of high *Z*, NP aggregation is able to produce hot spots of dose enhancement.^[^ ^178^ ^]^ The dose enhancement is LET‐dependent, being higher at low X‐ray energies,^[^ ^179^, ^180^ ^]^ such LET‐dependent radiosensitization with Au NPs has been reported by Li et al.^[^ ^181^ ^]^

### 4.2 ROS Generation at Nanoparticle Surfaces by Light or Ionizing Radiation

The second important effect is ROS generation at the NP surface. For example, light excitation of inorganic NPs such as TiO_2_, ZnO or Si at photon energies above the bandgaps of 3.2 eV (TiO_2_), 3.36 eV (ZnO), and 1.4 eV (Si) produces electron/hole pairs that induce a series of ROS‐generating reactions in the aqueous component. For example, small (<5 nm) Si NPs generate 10 × 10^−6^ m of ROS ($O_{2}^{-}$/${\mathrm{HO}}_{2}^{•}$, HO^•^, and H_2_O_2_) per Gy in a 6.4 × 10^−6^ m aqueous solution of Si NPs under 4 MeV X‐ray radiation,^[^ ^182^ ^]^ which translates to 2.5 × 10^8^ ROS per Gy in a sphere of 10 µm radius, comparable in size to a cell. Assuming a typical clinical dose rate of 200 cGy min^−1^,^[^ ^183^ ^]^ the generation rate is 8 × 10^5^ ROS per second per cell, compared to the basal metabolic rate of ≈10^5^.^[^ ^82^ ^]^

The yields of specific radiolysis products at solid/liquid interfaces can be significantly higher than in bulk water,^[^ ^57^ ^]^ so that the ROS generated by a given radiation dose can be distinctly higher in the presence of NPs than without.^[^ ^99^, ^184^ ^]^ For example, Cho quantified this enhancement for different Au NP concentrations (7, 18, and 30 mg/1 g of tissue) by Monte Carlo calculations for 140 kVp, 4 and 6 MV photon beams, and ^192^Ir gamma.^[^ ^165^ ^]^ The average dose enhancement over the tumor volume in this work was 2.0 and 5.6 in the presence of 7 mg /1 g and 30 mg /1 g of Au inside the tumor for 140 kVp X‐rays. The dose enhancement was much lower in the case of 4 MV X‐rays (1.009 and 1.032 for 7 mg/1 g and 30 mg/1 g of Au) and 6 MV X‐rays (1.007 and 1.025 for 7 mg/1 g and 30 mg/1 g of Au). These data provide a clear indication that clinically significant tumor dose enhancement can only be achieved with low energy X‐ray photons.

**Figure 5:**
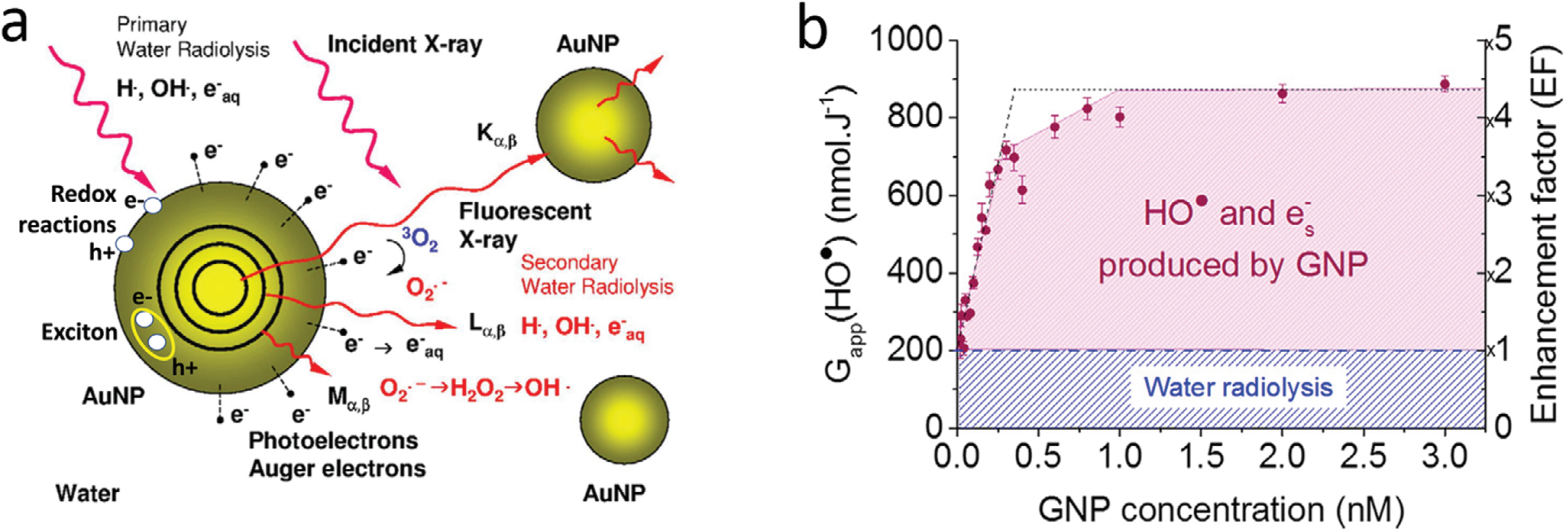
a) Interactions of X‐rays with NPs in aqueous solution (note that some of these reactions require dissolved oxygen). Reproduced with permission.^[^ ^184^ ^]^ Copyright 2011, Elsevier. b) Hydroxyl and hydrated electron yields produced by Au NPs during radiotherapy and the corresponding ROS enhancement factor. Reproduced with permission.^[^ ^99^ ^]^ Copyright 2018, Elsevier.

There are several possible mechanisms for ROS generation in NPs. One example^[^ ^99^ ^]^ is illustrated in **Figure** **5**a where we highlight the secondary electrons with energies higher than the work function of the material (5.2 eV for Au).^[^ ^184^ ^]^ These electrons are then released from the NP to the solution, where subsequent reactions produce ROS. An alternative mechanism proceeds through generation of excited electron–hole pairs in the NPs (Figure 5a). The pairs that do not rapidly recombine provide electrons and holes that individually may escape the NP and undergo redox reactions in the solution.^[^ ^100^ ^]^ Finally, an excitonic effect may take also place,^[^ ^185^ ^]^ where the energy of the bound‐electron hole pair (exciton) is transferred out from the NP to the solution where it facilitates ROS generation (Figure 5a). The net effect of these processes is generation of ROS that is over and above the amounts generated by radiation in the absence of NPs (Figure 5b). The opposite effect, namely ROS scavenging by NPs, has also been shown. For example, CeO_2_ NPs are efficient scavengers of ROS such as HO^•^ and RNS such as NO^•^.^[^ ^186^ ^]^

#### 4.2.1 Similarities between Photocatalysis and Radiocatalysis

Having established that ionizing radiation interacts with many types of NPs to generate ROS,^[^ ^187^ ^]^ we now consider how to increase ROS generation from the available radiation dose. One approach is to draw analogies between photocatalysis and radiocatalysis in the presence of solid surfaces that may lower the activation energies. Although the initial physical effects are different, they lead to the same outcome, with the energy of light or radiation dispersed into heat and, under specific conditions, electron emission, and excitation of electron–hole pairs. As a result of this similarity, many aspects of radiocatalysis and photocatalysis are qualitatively similar, allowing insights from photocatalysis to explain radiation‐induced processes. For example, TiO_2_ NPs are excellent photocatalysts for ROS generation, particularly when coated with Au, Ag or Pt,^[^ ^188^, ^189^, ^190^ ^]^ suggesting that TiO_2_ NPs may be effective radiosensitizers, as reported.^[^ ^191^ ^]^ Excitation of inorganic NPs such as TiO_2,_ ZnO or Si at photon energies exceeding their bandgaps of 3.2, 3.36, and 1.4 eV, respectively produces electron/hole pairs that induce a series of chemical reactions in aqueous solution to generate ROS.^[^ ^192^ ^]^ It could, therefore, be expected that both pure ZnO and SiO_2_‐coated ZnO NPs would serve as sensitizers in radiation‐induced PDT. Indeed, it has been demonstrated that the cell kills by such NPs in human prostate adenocarcinoma cell lines was increased by a factor of 1.5–2 compared to radiation alone.^[^ ^193^ ^]^

**Figure 6:**
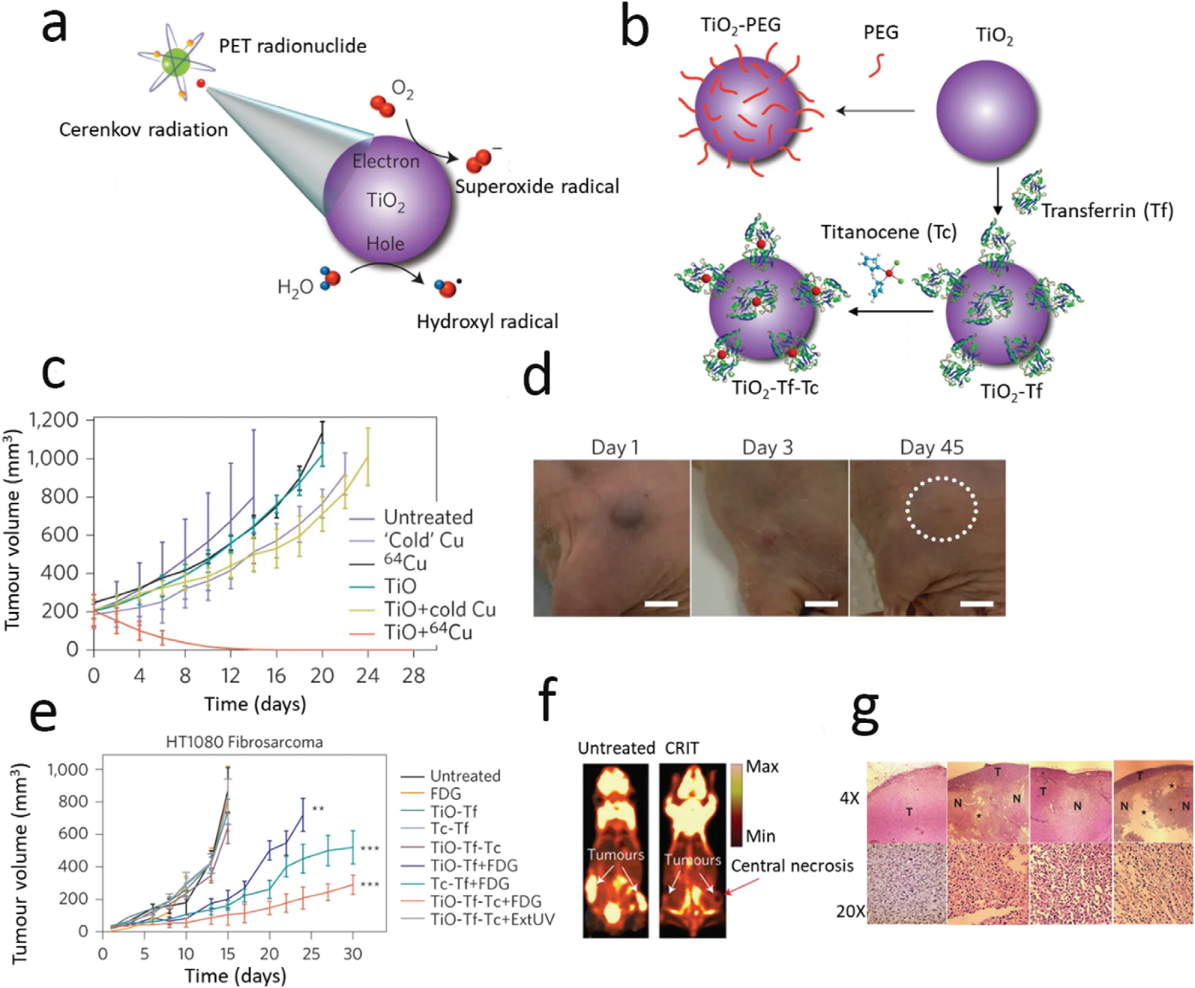
a) Schematic of CR‐mediated ROS generation from TiO_2_ NPs. b) Schematic of TiO_2_–PEG, TiO_2_–Tf, and TiO_2_–Tf–Tc synthesis. c) HT1080 tumor growth in mice using the NPs and ^64^Cu, together with controls. b) Reduction in tumor size at 1, 3, and 45 d post treatment (single dose of 2.5 µg mL^−1^ TiO_2_–PEG and 0.5 mCi/0.1 mL of ^64^Cu). e) Tumor growth following a single intratumoral administration of TiO_2_–Tf–Tc with FDG radionucleotide, together with controls. f) FDG‐PET imaging of untreated (left) and treated (right) mice with bilateral HT1080 tumors at 15 and 30 d, respectively. g) H&E stained treated and control tumor sections at two different magnifications (T‐tumor, N‐necrotic *‐denuded areas indicating macrophage‐assisted tumor cell clearance). Reproduced with permission.^[^ ^198^ ^]^ Copyright 2015, Springer Nature.

ROS generation under X‐ray exposure may also be partly attributed to CR light‐induced PDT, although this is limited by the number of light photons generated at clinical X‐ray doses.^[^ ^70^ ^]^ CR‐mediated PDT has been reported,^[^ ^194^, ^195^, ^196^, ^197^, ^198^, ^199^ ^]^ for example, with TiO_2_ NPs,^[^ ^198^, ^200^ ^]^ in conjunction with radiolabeled 2′‐deoxy‐2′‐(^18^F) fluoro‐d‐glucose (FDG) (half‐life: 1.83 h, *β* ^+^: 0.633 MeV, 97%) and ^64^Cu (half‐life: 12.7 h, *β* ^+^: 0.653 MeV, 19%, *β* ^−^: 0.579 MeV, 39%) used to generate CR. In this work, FDG was preferentially metabolized by tumors (human fibrosarcoma, HT 1080), allowing tumor‐specific targeting and CR generation. The NPs were conjugated with apo‐transferrin (Tf) for tumor targeting, while additional conjugation of titanocene (Tc), a photoinitiator in the metallocene family, enhanced and complemented the cytotoxicity of TiO_2_ (**Figure** **6**a–g). A significant shrinkage of the tumor volume (40% ± 5% within three days) and a complete tumor regression by 30 days was achieved in this work. This result indicates that CR mediated ROS generation may be sufficient for an effective treatment of deep‐seated tumors.

#### 4.2.2 Charge Transfer Photo‐ and Radiocatalysis‐Redox Reactions

**Figure 7:**
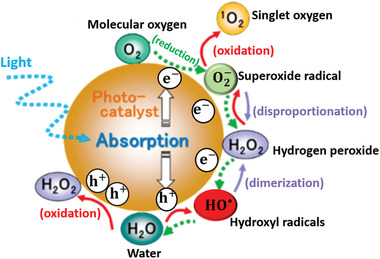
Reactive oxygen species generated in photocatalysis at the NP surface in oxygenated aqueous environment. Reproduced with permission.^[^ ^100^ ^]^ Copyright 2017, American Chemical Society.

Solid surfaces in an aqueous environment may catalyze light‐ and radiation‐induced ROS‐generating redox reactions,^[^ ^100^, ^201^, ^202^ ^]^ hence understanding the photocatalytic redox reactions aids in optimizing ROS yields in generally less‐well explored radiocatalysis. ROS may be created from both H_2_O and any available O_2_ by consecutive reactions. HO^•^, H_2_O_2_, $O_{2}^{-},$ and ^1^O_2_ are generated by stepwise oxidation of H_2_O, while stepwise reduction of O_2_ generates $O_{2}^{-}$, H_2_O_2_, and HO^•^, (**Figure** **7**), as for example detailed by Nosaka and Nosaka.^[^ ^100^ ^]^

**Figure 8:**
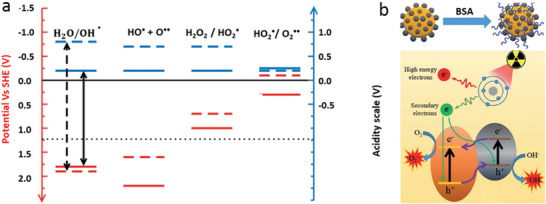
a) Energy level diagram for the steps in water oxidation: red – electronic, blue – protonic. Solid lines represent levels calculated on TiO_2_; dashed lines are experimental levels in aqueous solution. Black arrows – dehydrogenation potentials, dotted black line – standard redox potential of the overall water oxidation reaction. Reproduced with permission.^[^ ^207^ ^]^ Copyright 2014, Wiley‐VCH. b) Mechanism by which BSA‐coated BiOI@Bi_2_S_3_ semiconductor heterojunction NPs enhance the efficiency of ROS generation by ionizing radiation. Reproduced with permission.^[^ ^202^ ^]^ Copyright 2017, Wiley‐VCH.

The basic mechanism of light‐ or radiation‐mediated redox reactions is illustrated in **Figure** **8**a, where an absolute potential of the standard hydrogen electrode of −4.42 eV^[^ ^203^ ^]^ was used to align the conduction and valence band edges with the values of the redox potential. Most of the excited (thermalized) electrons and holes rapidly recombine (90% within 10 ns in TiO_2_^[^ ^204^ ^]^) but some can tunnel out of the solid to participate in redox reactions in the water at the NP surface. For the reduction reaction to occur, the conduction‐band edge of the solid surface must be located above, i.e., be more negative than, the potential of the (oxidized) acceptor species to make it energetically favorable for the excited electron to migrate out of the solid. Likewise, when the valence band edge of the solid surface is below, i.e., more positive than, the potential of the donor (reduced) species, then it is energetically favorable for the photoexcited hole to migrate out of the NP.^[^ ^202^ ^]^ Charge neutrality must be maintained in this process, so that for each charge vacating the solid the opposite charge must leave the solid. This process may be facilitated by electron/hole scavenger compounds such as ascorbic acid conjugated to or adsorbed at the NP surface.^[^ ^205^, ^206^ ^]^ If such scavengers are not provided, then the NPs undergo chemical degradation or, for example, may chemically transform the adsorbed ligands, leading to NP aggregation.^[^ ^205^ ^]^ Close alignment of the conduction band and/or valence band edges with the reduction and/or oxidation potentials is desirable for efficient reduction and/or oxidation.^[^ ^202^, ^207^ ^]^ It should be noted, however, that these considerations do not impact the reaction rates, which are determined by activation energies and concentrations.

As an example, TiO_2_ in aqueous suspension generates ROS upon irradiation with light of sufficient energy (>3.2 eV) to produce electron–hole pairs.^[^ ^204^ ^]^ These electrons and holes are able, respectively, to take part in reduction and oxidation reactions, with redox potentials shown in Figure 8a. These potentials are strongly influenced by the presence of the TiO_2_ surface^[^ ^207^ ^]^ and depend on the solution pH, as described by the Nernst equation.^[^ ^121^, ^208^ ^]^ The ROS photoproducts on the TiO_2_ surface are reported to be $O_{2}^{-}$, HO^•^ radicals, and H_2_O_2_, created in the following reactions^[^ ^204^ ^]^

(13) $$\begin{array}{c} O_{2}+e^{-}\to O_{2}^{-} \end{array}$$

The HO^•^ generation can be increased by adding H_2_O_2_ to the NP solution, according to

(14) $$\begin{array}{c} H_{2}O_{2}+e^{-}\to HO^{•}+OH^{-} \end{array}$$

(15) $$\begin{array}{c} H_{2}O_{2}+O_{2}^{-}\to HO^{•}+OH^{-}+O_{2} \end{array}$$

The hydroxyl ions are also generated from water but at low yield. This process involves the oxidation of OH^−^(H_2_O) by photogenerated holes in the valence band ($h_{\mathrm{vb}\;}^{+})$

(16) $$\begin{array}{c} h_{\mathrm{vb}}^{+}+OH^{-}\to HO^{•} \end{array}$$

(17) $$\begin{array}{c} h_{\mathrm{vb}}^{+}+H_{2}O\to HO^{•}+H_{\mathrm{aq}}^{+} \end{array}$$

It is also worth noting that electron acceptors (such as compounds containing, e.g., Fe^3+^) are capable of stimulating HO^•^ generation, as they may inhibit electron–hole recombination in the solid and thus facilitate the interaction of holes with water.^[^ ^204^ ^]^ Such electron acceptors can be incorporated as part of the NP design.

#### 4.2.3 Heterojunctions and Carrier Storage in Charge Transfer Photo‐ and Radiocatalysis

The photo‐ and radiocatalytic properties of NPs may be optimized to ensure high ROS yields by reducing the probability of recombination of the excited electron–hole pairs. A heterojunction approach ensures that these charge carriers can be more effectively utilized in redox reactions with charge transfer out of the NPs. For example, trapping electrons in a semiconductor NP combined with a metal such as Au has been used to induce charge separation.^[^ ^209^ ^]^ Photocatalytic semiconductor NPs based on the application of heterojunctions for enhanced ROS generation have been also proposed^[^ ^202^ ^]^ and photocatalytic NPs based on bismuth oxyiodide (BiOI) have been explored as a radiosensitizer in X‐ray excited PDT, where the radiosensitization was enhanced by coating the BiOI surface with Bi_2_S_3_ to form heterojunction NPs with low electron–hole pair recombination energy (Figure 8b). This work used a “sandwich” geometry so that both sides of the NP‐embedded heterojunctions are exposed to water, enabling opposite migration of electrons and holes. An alternative strategy, implemented^[^ ^210^ ^]^ is to ensure that one type of charge is captured in the material, allowing the opposite charge to take part in the redox reactions unhindered by the Coulomb attraction to its counterpart. An important prerequisite for this is long electron–hole pair lifetime, so that it is beneficial to reduce the electron–hole recombination rate, for example, by facilitating the formation of triplet exciton states.^[^ ^205^ ^]^

#### 4.2.4 Resonant Effects in Energy‐Transfer Photocatalysis

**Figure 9:**
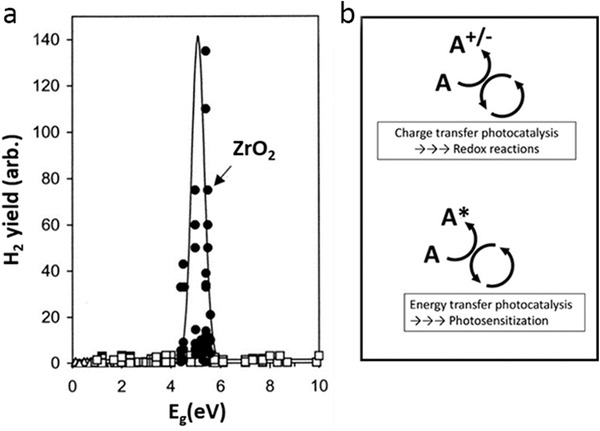
a) Yields of molecular hydrogen as a function of oxide bandgap in radiolysis of water adsorbed on various oxides. Reproduced with permission.^[^ ^185^ ^]^ Copyright 2001, American Chemical Society. b) Charge transfer photocatalysis involving redox processes.

The modification of ROS yields in redox reactions discussed above suggests a range of potential optimization strategies, although none provide high amplification. However, resonance phenomena uncovered in research on photocatalytic water splitting^[^ ^185^ ^]^ promise a much higher enhancement. For example, an ≈100‐fold increase in hydrogen yield was reported on solid surfaces with a bandgap ≈5 eV that is close to (resonant with) the energy of the H—OH bond in water (5.1 eV) (**Figure** **9**a),^[^ ^185^ ^]^ which was attributed to energy transfer from excitons to the H—OH bond,^[^ ^211^ ^]^ a mechanism referred to as “energy transfer photocatalysis,” as reviewed by Strieth‐Kalthoff et al.^[^ ^212^ ^]^

In general, energy transfer is a photochemical process where a donor molecular entity in an excited state (D*) is deactivated to a lower state (D) by transferring its energy to an acceptor (A) to produce the excited state (A*)

(18) $$\begin{array}{c} D^{*}+A\to D+A^{*} \end{array}$$

This is distinct from charge‐transfer photocatalysis that involves redox processes, where charges rather than energy are transferred (Figure 9b). Energy transfer is mediated by Coulomb dipole–dipole and exchange interactions and it typically does not involve photon transfer, so that A does not need to have appreciable optical absorption for this process to be effective. Generation of singlet oxygen in PDT is an example of energy‐transfer photocatalysis. The energy transfer is facilitated if D* is long‐lived (>100 ns), as in triplet molecular states. Strong excitonic effects lead to low yields of free charge carriers and limit the mobility of charge carriers, which increases the quantum yield of energy‐transfer photocatalysis.^[^ ^211^ ^]^

Energy‐transfer often exhibits resonant behavior, as seen for example in plasmonic resonances.^[^ ^213^, ^214^, ^215^ ^]^ The energy transfer photocatalysis mechanism is relevant for ROS generation, for example, in the novel material, 2D black phosphorus,^[^ ^211^ ^]^ where the close valence band edge and redox potential enables effective water oxidation and the formation of hydroxyl ions. Excitonic effects in energy‐transfer photocatalysis have been demonstrated to increase photocatalytic yields.^[^ ^210^ ^]^ For example, the closeness of the excitonic‐state energy and the ^1^O_2_ conversion energy enables effective generation of ^1^O_2_.^[^ ^216^ ^]^ The presence of excitonic resonances in photocatalysis suggests the possibility of resonantly enhanced ROS yield in radio‐catalysis as well, and we propose that this could be achieved by carefully tuning NP systems through bandgap engineering, doping or the application of novel low‐dimensional materials.

#### 4.2.5 Effects of Surface Functionalization and Coatings on ROS Generation

NP surface properties such as interfacial layers, coatings, functionalization, and/or capping agents can play a significant role in ROS generation.^[^ ^217^, ^218^ ^]^ They can mediate chemical reactions, for example, in processes where electrons and/or holes generated in the NPs interact with the ligands before they are released into solution and take part in ROS‐generating redox reactions. These intermediate steps may change the ROS yields and kinetics as, for example, in adsorption of O_2_ at the surface of AuNPs, which disrupts water organization at the NP surface and reduces the radical production rates.^[^ ^99^ ^]^

**Figure 10:**
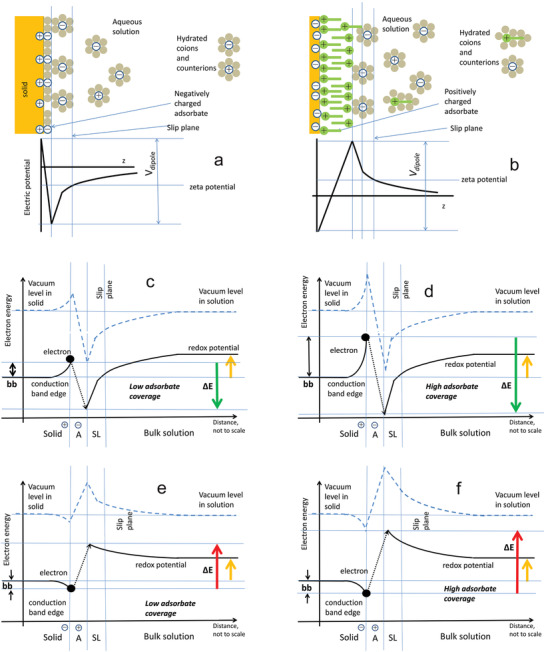
Effect of surface adsorbate on redox energy for a reaction where an electron leaves the solid surface to form a radical species. a,b) Ionic and electric potential in the vicinity of a solid surface in the modified Stern model for (a) negatively charged surface and positively charged adsorbate (negative zeta potential) and b) positively charged surface and negatively charged adsorbate (positive zeta potential). c–f) Conduction band edge and redox potential in the presence of surface charges in solid charged adsorbate molecules and solution ions. The yellow arrow indicates the redox potential value (identical in panels c–f). Arrows labeled ∆*E* indicate positive (red) or negative (green) energies required for the electron (black dot) to take part in the redox reaction as marked. (The possibility of electron transfer to the adsorbate has not been noted here.) Note that the values of ∆*E* vary from positive to negative depending on the surface concentration of adsorbate and its charge. The vertical axis reflects the distance from the solid surface; distances are not to scale—the band bending in solids occurs on a µm scale, while the width of the adsorbate and distance from solid surface to the slip plane are within nm. The electron is assumed to be located at the solid surface, reflecting its location in an NP. Valence and other band edges in the solid have been omitted for clarity. (a,b) Adapted with permission.^[^ ^220^ ^]^ Copyright 2012, Wiley‐VCH.

The surface charge of NPs solids, as reflected by their zeta potential, ensures their colloidal stability by changing the energy‐level structure of the solid–liquid system, since the Coulomb energy from the additional charge combines with the electronic energy to increase the total electron energy. The surface charge density also produces band bending (**Figure** **10**a,b) and leads to the organization of ions and coions in solution according to the Gouy–Chapman–Stern theory.^[^ ^219^ ^]^ The interfacial water layer itself is also organized.^[^ ^99^ ^]^ The presence of charged adsorbates at the solid–liquid interface additionally modifies the arrangement of surface charges and leads to the potential profiles illustrated in Figure 10a,b, as a result of which the aligned vacuum levels undergo spatial modification near the interface. Other significant energy levels, such as conduction and valence band edges as well as redox potentials, follow the spatial variations of electrical potential according to the energy addition principle, and lead to alterations of the energy required for electrons to take part in redox reactions (Figure 10c–f). This energy varies from negative (Figure 10d) to positive (Figure 10f) as a result of changes in the surface charge density and polarity. The former can be easily engineered by modifying the NP coating/functionalization,^[^ ^220^ ^]^ which offers a potentially simple way to improve the alignment of conduction and valence band edges with the redox potentials in water. Thus, surface phenomena such as the surface charge, interfacial organization of molecular species, and capping and/or functionalization can be exploited to optimize ROS generation. The published literature provides numerous examples that the surface chemistry of NPs, and properties such as surface charge are able to affect the amount of ROS generated by NPs.^[^ ^221^, ^222^ ^]^ For example, positively charged Si NP‐NH_2_ proved to be more cytotoxic in terms of reducing mitochondrial metabolic activity and effects on phagocytosis than neutral Si NP‐N3 because positively charged Si NP‐NH_2_ were found to produce the highest level of intracellular ROS.^[^ ^223^ ^]^ Iron oxide NPs coated with positively charged chitosan were reported to produce abundant ROS, underpinning their significant antimicrobial activity against *Escherichia coli* and *Bacillus subtilis*.^[^ ^224^ ^]^ High surface charge on carbon nanotubes has been found responsible for the prooxidant effects of CNT.^[^ ^225^ ^]^ It was also observed that increasing the hydrophobicity of the AuNPs increased their cytotoxicity, and increased ROS production.^[^ ^226^ ^]^ Similar effects have been reported for the generation of hydroxyl radicals during radiolysis of water in the presence of AuNPs.^[^ ^99^ ^]^ Another example of the effect of NP surface coating on ROS generation is decreased emission rate of electrons from PEG‐coated AuNPs with increasing PEG layer thickness, as well as altered secondary electron energy spectrum,^[^ ^205^ ^]^ both of which impact the ROS yield.

#### 4.2.6 Effects of Nanoparticle Size and Shape on ROS Generation

The effects of NP size and shape on ROS generation upon radiation exposure have been widely observed.^[^ ^101^ ^]^ They also offer scope for tuning the ROS yields. For example, the ROS generation rates are higher in smaller NPs due to the larger surface area to mass ratio, combined with a linear dependence of the ROS generation rate on the surface area, as reported for example for AuNPs.^[^ ^184^ ^]^ Other authors have also suggested a more efficient electron emission from smaller NPs, since the excited electrons have a lower probability of dissipating their energy inside the NP before reaching the surface.^[^ ^227^ ^]^

The effects of NP shape on ROS generation, also widely observed,^[^ ^228^ ^]^ and they can be attributed to a different mix of crystalline orientations with varying chemical activity. The effect of this orientation on the emission of electrons and reactive species generation is well established, for example, in water splitting by atanase and rutile TiO_2_ NPs whose crystalline surface ordering differ.^[^ ^229^ ^]^ Different orientations of nanocrystal facets yield sufficiently variable chemical properties that fully anisotropic facet‐dependent functionalization has been realized.^[^ ^230^ ^]^ Tuning the NP shape offers a viable strategy to optimize ROS yields, especially if theoretical estimates of favorable surface energetics are available.

### 4.3 Generation of Light from Scintillating Nanoparticles Exposed to Ionizing Radiation

Scintillation refers to generation of light from materials exposed to ionizing radiation.^[^ ^231^, ^232^, ^233^, ^234^ ^]^ In solid materials, this begins with the creation of excited electron–hole pairs which migrate through the material with some energy loss^[^ ^235^ ^]^ or become trapped at specific defect centers where their radiative recombination generates light.^[^ ^231^ ^]^ Many materials, mostly organic and inorganic solids, have scintillation properties. Inorganic scintillators such as CeF_3_, YAG, BGO, LaCl_3_(Ce), LaBr_3_(Ce) are perhaps the best known and are employed in radiation detectors.^[^ ^232^, ^236^ ^]^ NPs from these materials can be readily prepared. Scintillating NPs (SNPs) are able to act as energy transducers for ionizing radiation and this can be exploited for X‐ray induced generation of ROS from conjugated PSs.^[^ ^32^, ^38^, ^180^, ^237^, ^238^, ^239^, ^240^, ^241^ ^]^ Many SNPs emit light in the UV–vis region in response to ionizing radiation^[^ ^180^, ^238^ ^]^ and can perform as nanoscale light sources within the tissue, where they generate cytotoxic ROS.^[^ ^37^ ^]^ Kamkaew et al. ^[^ ^32^ ^]^ provide an excellent review of this topic.

### 4.4 Free Radical Scavenging by Nanoparticles

While this review centers on enhancing radiotherapy with engineered nanomaterials increasing the oxidative stress at the therapy site, it is worth mentioning that mitigation of this stress in adjacent healthy tissue also has a significant clinical value—despite the well‐developed technique of radiotherapy planning. The application of NPs for radioprotection has been discussed in^[^ ^242^, ^243^ ^]^ where key classes of nanomaterial radioprotectors are listed. These include encapsulated molecular radioprotectors such as flavonoids^[^ ^244^ ^]^ or other compounds formulated in liposomes,^[^ ^245^ ^]^ polysaccharides,^[^ ^246^ ^]^ fullerenes, carbon NPs of various types^[^ ^247^, ^248^ ^]^ some of which are clinically approved as well as inorganic ceria (CeO_2_).^[^ ^249^ ^]^ The latter represents a catalytic system with interesting enzyme‐mimetic properties able to reduce oxidative and also nitrosative stress^[^ ^186^ ^]^ mostly related to the of the Ce^3+^/Ce^4+^ valence ratio on the NP surface. This area merits further investigation as ceria under certain conditions was found to differentially protect normal cells without affecting cancer cells.^[^ ^250^, ^251^, ^252^ ^]^ However, under alternative surface conditions ceria was found to enhance the effects of radiation.^[^ ^253^, ^254^ ^]^

In order to fully realize the therapeutic benefit of protecting healthy tissue unduly exposed to radiation the free‐radical scavenging nanomaterials would need to be targeted to this healthy tissue which represents a major problem. Targeting whole organs would be an important, but extremely challenging first step. Preferential renal or hepatic uptake has been demonstrated^[^ ^255^, ^256^ ^]^ facilitated by special physiology of these two organs. Organ‐specific drug delivery has been reviewed,^[^ ^257^ ^]^ and it remains an active area of research.

## 5 Interaction of Radiation with Photosensitizers: Physical and Chemical Mechanisms

NP designs used in X‐PDT may incorporate molecular PSs. These generate additional free radicals (e.g., ^1^O_2_) beyond the levels produced by water radiolysis. Many common dyes, including methylene blue, crystal violet, phthalocyanine, and heptamethine, are efficient PSs.^[^ ^32^, ^258^ ^]^ Some PSs, such as riboflavin or porphyrins, are endogenous to cells.^[^ ^259^, ^260^ ^]^ Clinically approved exogenous PSs include tetrapyrrole structured hematoporphyrin derivative (HpD), Photofrin,^[^ ^261^, ^262^, ^263^ ^]^ 5‐Aminolevulinic Acid (5‐ALA) that leads to exogenous production of protoporphyrin IX (PpIX),^[^ ^264^ ^]^ verteporfin (and its liposomal clinical formulation, Visudyne)^[^ ^239^, ^265^ ^]^ and palladium bacteriopherophorbide, TOOKAD.^[^ ^266^, ^267^ ^]^ A range of other PSs including zinc phthalocyanine, aluminum phthalocyanine tetrasulfonate, and lutexaphyrin are currently undergoing clinical trials.^[^ ^268^, ^269^, ^270^ ^]^ Recently discovered PSs based on aggregation‐induced emission (AIE) molecules offer enhanced ^1^O_2_ yields.^[^ ^271^ ^]^ For example, 80% yield was reported for an oligo‐ethyleneimine (OEI)‐crosslinked polycation, compared to 28% for the clinical Photofrin^[^ ^272^ ^]^). This effect is due to restricted nonradiative decay channels in the AIE systems.^[^ ^273^ ^]^ Conversely, PSs activated by near‐infrared light (which is advantageous due to increased penetration depth in tissue) have reduced yields of cytotoxic species due to their comparatively high nonradiative transition rates.^[^ ^32^ ^]^ Additionally, both exogenous, but also endogenous PSs in cells (e.g., riboflavin) exposed to light or to ionizing radiation may, in principle, be able to change the balance of cellular ROS. It is, therefore, important to understand the underlying molecular processes.

### 5.1 Interaction of Visible Radiation with Photosensitizers

Singlet oxygen generation by a PS is initiated by its excitation with a photon of light that is spectrally matched to the absorption by relevant energy levels in this molecule. The resulting excited singlet state (PS^E^) may then undergo intersystem crossing to an excited triplet state (PS^T^), which has a comparatively long lifetime (10^−9^–10^−6^ s).^[^ ^274^ ^]^ The triplet state may then return to the ground state (PS^G^) by initiating a photochemical reaction, leading to the generation of ROS^[^ ^275^, ^276^, ^277^ ^]^ through type I and/or type II reactions.

**Figure 11:**
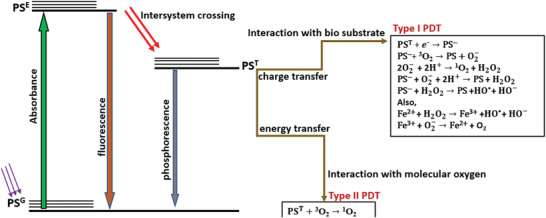
Jablonski diagram showing the possible events after a PS molecule has been excited. Type I reaction occurs as a result of charge transfer and it may lead to the production of superoxide anion, hydrogen peroxide, and hydroxyl radicals, whereas the Type II reaction produces singlet oxygen via energy transfer to ground‐state oxygen. PS^G –^ ground state PS; PS^T^‐triplet state PS, and PS^−^‐reduced state PS. Adapted with permission.^[^ ^96^ ^]^ Copyright 2016, Elsevier.

In type I reactions, the electron transfer from PS^T^ to the surrounding biomolecules (substrate) generates free radicals that react with available oxygen producing superoxide radical anions. Further addition of a proton can lead to the formation of hydrogen peroxide (H_2_O_2_) or biologically reactive hydroxyl radicals.^[^ ^278^ ^]^ Alternatively, in type II reactions, PS^T^ can transfer energy directly to ground‐state molecular oxygen (^3^O_2_) to generate ^1^O_2_.^[^ ^276^, ^279^ ^]^ **Figure** **11** illustrates these reaction pathways and types of ROS generated. Type I and type II reactions can occur simultaneously, their relative contributions depending on the oxygen concentration and the interacting biomolecules. The effectiveness of ROS and ^1^O_2_ generation critically depends on the availability of molecular oxygen.^[^ ^280^, ^281^ ^]^

Singlet oxygen generation by a PS is initiated by its excitation with a photon of light that is spectrally matched to the absorption by relevant energy levels in this molecule. The resulting excited singlet state (PS^E^) may then undergo intersystem crossing to an excited triplet state (PS^T^), which has a comparatively long lifetime (10^−9^–10^−6 ^s).^[^ ^271^ ^]^ The triplet state may then return to the ground state (PS^G^) by initiating a photochemical reaction, leading to the generation of ROS^[^ ^272^, ^273^, ^274^ ^]^ through type I and/or type II reactions.

In type I reactions, the electron transfer from PS^T^ to the surrounding biomolecules (substrate) generates free radicals that react with available oxygen producing superoxide radical anions. Further addition of a proton can lead to the formation of hydrogen peroxide (H_2_O_2_) or biologically reactive hydroxyl radicals.^[^ ^275^ ^]^ Alternatively, in type II reactions, PS^T^ can transfer energy directly to ground‐state molecular oxygen (^3^O_2_) to generate ^1^O_2_.^[^ ^273^, ^276^ ^]^ Figure 11 illustrates these reaction pathways and types of ROS generated. Type I and type II reactions can occur simultaneously, their relative contributions depending on the oxygen concentration and the interacting biomolecules. The effectiveness of ROS and ^1^O_2_ generation critically depends on the availability of molecular oxygen.^[^ ^277^, ^278^ ^]^

### 5.2 Interaction of X‐Rays with Photosensitizers

PSs have been shown to generate ROS upon exposure to X‐rays, in particular porphyrin‐based molecules such as HpDs, Verteporfin, protoporphyrin IX, and Photofrin II.^[^ ^282^, ^283^ ^]^ The mechanisms have not been fully established, but several putative explanations have been put forward. For example, the initial excitation to the singlet state PS^E^ may be possible simply by interaction of the PS molecule with radiation‐induced secondary electrons^[^ ^284^, ^285^ ^]^ that generate molecular excitations, in a process analogous to cathodoluminescence.^[^ ^286^ ^]^ It is also worth mentioning that high energy X‐rays beyond the Cherenkov energy threshold can produce Cherenkov photons (CL). These, in turn, can excite the PS and generate highly reactive ^1^O_2_ via the conventional photodynamic pathways as in Figure 11.

## 6 Example Nanoparticle Designs for X‐PDT

The advancement of nanotechnology in the medical field opens a range of possibilities to improve therapeutic efficiency, especially by combining multiple approaches such as radiation and photosensitization for cancer treatments.^[^ ^287^ ^]^ The mechanism of ROS generation from NPs and PSs under light and X‐ray radiation and the active role played by the NPs may vary. The NPs can either act as a scintillator to produce UV–vis light to activate the PS, or act as a radiosensitizer or as a carrier for a radiosensitizing drug. In addition, the EPR effect and targeting possibilities further improve the efficiency of NP formulated therapeutics. In this section, we will consider the different roles of NPs in the context of X‐PDT and their therapeutic potential, particularly to treat deep‐seated tumors.

### 6.1 Scintillating Inorganic Nanoparticles Combined with Photosensitizers

When scintillation NPs are integrated with PSs, the ROS generated upon X‐ray exposure either directly by excitation of the PS, or indirectly via the scintillation light from the NPs or via the CL generated in the tissue. This raises the question whether combining PSs with NPs (either scintillating or not) leads to additional ROS generation from a given radiation dose. This question is not yet fully answered, but a theoretical study based on Monte Carlo simulation shed some light in the efficiency of ROS generation from scintillating NPs and PS which depends on several parameters, including the distance between the SNP and PS, the X‐ray photon energy, and the concentration and size of the NP.^[^ ^288^ ^]^ This was illustrated in the case of Gd_2_O_3_ NPs where the total energy deposited in Gd_2_O_3_ NP and water following an interaction with a specific energy X‐ray photon was found to be different for different occupation ratios (NP/tissue volume ratio). For example, the interaction of 500 keV X‐rays with 10 nm Gd_2_O_3_ NP in a tumor at an occupation ratio of 2 × 10^−3^ deposits 1.1 keV of energy in Gd_2_O_3_ NP, and 173.5 keV in water, respectively. At a higher occupation ratio of 7 × 10^‐3^, the deposited energy in NP was 3.71 keV. However, when the NP size was increased to 100 nm at the same occupation ratio of 7 × 10^‐3^, the deposited energy was estimated 4.28 keV. These findings highlight the fact that a significant fraction of energy is deposited within the NPs despite the primary interaction occurring in the surrounding media and this influences ROS (in this case ^1^O_2_) generation.^[^ ^288^ ^]^

A detailed quantitative analysis of the effectiveness of conjugating PSs to scintillating NPs is provided by Clement et al.^[^ ^180^ ^]^ This work centers on scintillating CeF_3_ NP that produce UV light upon X‐ray excitation to activate verteporfin (VP) to produce ^1^O_2_. In this work, 60 Gy X‐ray generated 1.2 × 10^8^–2 × 10^9 1^O_2_ molecules per cell when the tissue contained a 5% volume fraction of CeF_3_‐VP conjugate. This is comparable to ^1^O_2_ concentration (≈5 ×  10^7^ molecules per cell) that results in 1/e clonogenic surviving fraction.^[^ ^289^ ^]^

Other scintillating NPs that have been used in X‐ray activated ROS generation include LaF_3_:Ce, ZnS:Cu, Co, Y_2_O_3_, Gd_2_O_2_S:Tb, and LiGa_5_O_8_:Cr.^[^ ^27^, ^239^, ^290^, ^291^, ^292^, ^293^ ^]^ For example, LaF_3_:Ce^3+^, an efficient scintillator with a strong emission peak at 520 nm has been reported to excite a PS, PpIX^[^ ^292^ ^]^ enabling the generation of cytotoxic ROS under X‐ray excitation. Recently, scintillating LiGa_5_O_8_:Cr NPs together with the PS 2,3‐naphthalocyanine (NC) loaded into mesoporous silica NPs (NC‐LGO:Cr@mSiO_2_) was also used for X‐PDT.^[^ ^293^ ^]^ Interestingly, the X‐ray excited luminescence from LiGa_5_O_8_:Cr is persistent, lasting for hours after radiation has ceased, and its spectrum falls into the far‐red wavelength range (≈720 nm). The tumor‐specific antibody Cetuximab was conjugated to the carboxylic group on the surface of these NPs and the in vivo treatment in an H1299 orthotopic tumor model in mice with 6 Gy X‐ray dose showed strong growth suppression compared to untreated controls. Another study also reported tumor responses using a scintillating copper–cysteamine NP complex (Cu–Cy) activated by X‐rays.^[^ ^294^, ^295^ ^]^ Interestingly, these NPs also produce ^1^O_2_ in the presence of microwave radiation, which may represent an alternative approach to treat deep‐seated tumors.^[^ ^296^ ^]^

**Figure 12:**
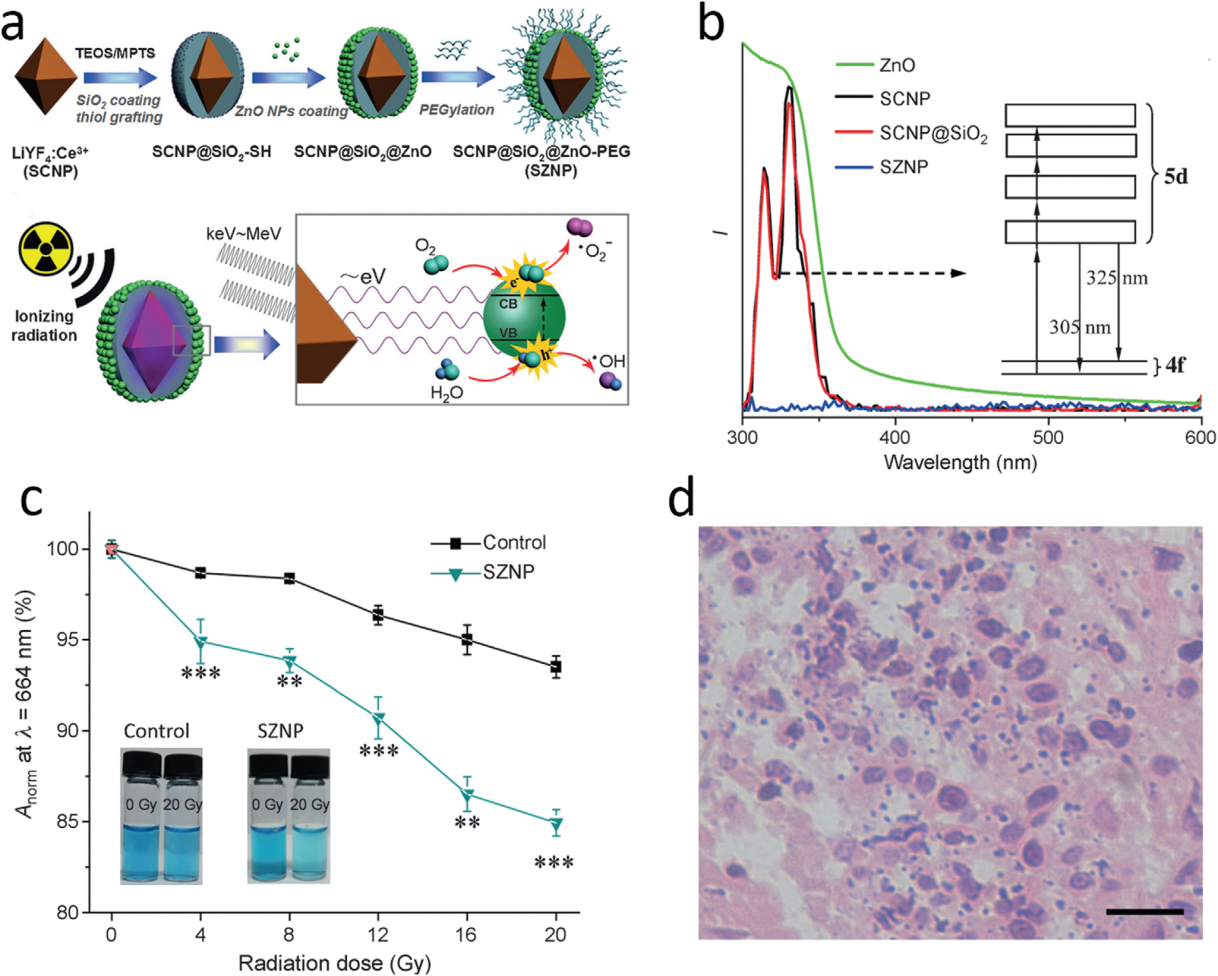
a) Synthetic route for monodispersed SZNPs and the mechanism of radiation induced PDT. b) Emission spectra of SCNPs, SCNP@SiO_2_ NPs under X‐ray excitation, UV–vis absorption spectra of SZNPs. The inset shows the energy‐level diagram of Ce^3+^ in LiYF_4_ crystal. c) ROS production as function of radiation dose indicated by change in MB absorption (*λ* = 664 nm) for SZNP and control solutions. d) H&E staining of tumor at 48 h after treatment with SZNP under 8 Gy radiation dose. Reproduced with permission.^[^ ^297^ ^]^ Copyright 2015, Wiley‐VCH.

A further example of a more complex inorganic nanostructure is SCNP@SiO_2_@ZnO‐PEG (SZNP) which brings together a nanoscintillator (LiYF_4_:Ce^3+^) coated with SiO_2_ (SCNP@SiO_2_) and a semiconductor NP (ZnO), with PEGylation for enhanced biocompatibility (**Figure** **12**a).^[^ ^297^ ^]^ The X‐ray excited emission shows strong peaks at 305 and 325 nm that match the absorption band of ZnO, facilitating effective energy transfer to generate hydroxyl radicals in the presence of water via a Type I pathway) (Figure 12b,c). This structure also shows a reduced oxygen dependence; in vitro experiments in HeLa cells suggesting that there is a comparable enhancement in ROS generation under normoxic and hypoxic conditions. The in vivo treatments showed marked reduction in tumors treated with SZNP and 8 Gy radiation (Figure 12d).

**Figure 13:**
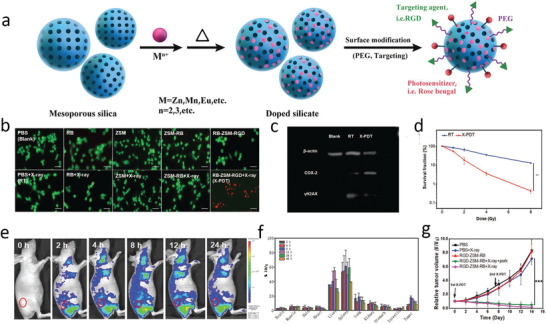
a) Formulation of RGD‐ZSM‐RB nanosensitizer. b) Live/dead cell imaging (green fluorescence for live cells, red fluorescence for dead cells) of U87MG cells with different treatments (Scale bar, 100 µm). c) Western blot assays confirming the effect of X‐PDT on DNA and membrane lipids. d) Clonogenic cell survival as a function of X‐ray dose. e) In vivo fluorescence images of tumor‐bearing mice (at 2, 4, 8, 12, and 24 h post injection i.v. of RGD‐ZSM‐RB. f) Biodistribution of RGD‐ZSM‐RB in main organs and tumor at different times after i.v. administration. g) Tumor growth curves (***: *P* < 0.001). Reproduced with permission.^[^ ^298^ ^]^ Copyright 2019, Wiley‐VCH.

A recent publication suggests that suitable metal dopants (Zn, Mn) in silicate act as nanoscintillators (ZSM) and produce visible X‐ray luminescence (450–900 nm);^[^ ^298^ ^]^ this luminescence then stimulates a conjugated PS, Rose Bengal (RB). This ZSM‐RB conjugate was conjugated to RGD peptide forming RB‐ZSM‐RGD that ensures high specific binding to the U87MG cells. The ROS generation process was effective enough to create sufficient ^1^O_2_ to kill U87MG human glioblastoma cells with 1 Gy of 50 KV X‐ray dose (**Figure** **13**a,b). The in vitro X‐PDT responses suggest that RB‐ZSM‐RGD is a potential candidate for treating glioblastoma. In vivo fluorescence imaging (Figure 13e) and the NP biodistribution (Figure 13f) confirm high accumulation of NP in cells (18% injected dose per gram) after 8 h (Figure 13f). Significant tumor reduction was shown in a U87MG model in mice with an intratumoral dose of 20 mg kg^−1^ and X‐ray activation (Figure 13g).

**Table** **3** lists inorganic nanoscintillators that have been reported for in vivo and/or in vitro X‐PDT. Organic scintillators for X‐PDT application remain largely unexplored.

**Table 3:** Summary of scintillating NPs used in X‐PDT

Nanoparticles (NPs)	Photosensitizer (PS)	Radiation conditions	Targeted (yes/no)	In vitro/in vivo	Ref
CaF_2_‐Tm	PPIX	100 kV, 1.5 Gy	No	In vitro	^[^ ^299^ ^]^
CeF_3_	Verteporfin (VP)	6 MeV, 6 Gy	No	In vitro	^[^ ^180^ ^]^
Gd_2_O_2_S:Tb	Photofrin II	130 kV, 20 mA, 15 min	No	In vitro	^[^ ^291^ ^]^
Gd_2_(WO_4_)_3_:Tb	Merocyanine 540 (MC540)	160 kV, 5–8 Gy	No	In vivo	^[^ ^300^ ^]^
GdEuC12 micelle	Hypericin (Hyp)	Synchrotron SOLEIL 400 mA, 10^15^ photons per second	No	In vitro	^[^ ^301^ ^]^
LaF_3_:Ce	Protoporphyrin IX (PPIX)	90 keV, 0.5 Gy min^−1^	No	In vitro	^[^ ^292^ ^]^
LaF_3_:Tb^3+^	Meso‐tetra(4‐carboxyphenyl) porphine (MTCP)	250 keV, 0.44 Gy min^−1^	No	In vitro	^[^ ^237^ ^]^
LaF_3_:Tb@ SiO_2_	Rose Bengal (RB)	75 kV, 20 mA	No	In vitro	^[^ ^302^ ^]^
LaF_3_:(Ce^3+^)/PLGA nanocomposite	PPIX	90 kV, 0.5 Gy min^−1^	No	In vitro	^[^ ^292^ ^]^
LiGa_5_O_8_:Cr (LGO:CR)@mSiO_2_	2,3‐naphthalocyanine	50 kV, 70 µA, 5 Gy	Yes: EGFR targeting	In vivo	^[^ ^293^ ^]^
LiLuF_4_:Ce@SiO2	Ag_3_PO_4_–Pt (IV)	6 MV, 4 Gy	No	In vivo	^[^ ^303^ ^]^
LiYF_4_@SiO_2_@ZnO	ZnO	220 keV, 2–8 Gy	No	In vitro	^[^ ^297^ ^]^
NaGdF4:Tb^3+^	RB	80 kV 0.5 mA, 20 min	No	In vivo	^[^ ^304^ ^]^
SrAl_2_O_4_:Eu^2+^ (SAO)@SiO_2_	MC540	50 kV, 70 µA, 0–5 Gy	No	In vivo	^[^ ^27^, ^39^ ^]^
SiC/SiO*_x_* nanowires (NWs)	H_2_TCPP	6 MV, 2 Gy	No	In vitro	^[^ ^305^ ^]^
Sr_2_MgSi_2_O_7_:Eu^2+^, Dy^3+^	PPIX	0–7 Gy	Yes: Folic acid targeted	In vitro	^[^ ^306^ ^]^
Tb_2_O_3_@ SiO_2_	Porphyrin	44 kV, 40 mA, 5.4 mGy s^−1^	No	In vitro	^[^ ^307^ ^]^
Y_2_O_3_	Psoralen (Ps)	2 Gy, 160 or 320 kVp	Yes: Nuclear targeted	In vitro	^[^ ^290^ ^]^
Zn‐ and Mn‐incorporated silica (ZSM)	RB	50 kV, 1.0 Gy	Yes: *α*_v_ *β*_3_ receptor targeting	In vivo	^[^ ^298^ ^]^
ZnS:Ag, Co	Tetrabromorhodamine‐123 (TBrRh123)	Faxitron RX‐650 cabinet	No	In vitro	^[^ ^239^ ^]^

### 6.2 Organic Nanoparticles Integrated with Photosensitizers

Advances in nanoscale drug formulations make it possible to produce organic NPs comprising molecular PSs.^[^ ^308^ ^]^ This approach is frequently required in standard therapeutic use of PSs, which typically have poor water solubility and low biocompatibility.^[^ ^309^, ^310^ ^]^ NP formulations combined with tumor‐targeting ligands allow, in principle, to enhance tumor selectivity of PSs and limit their toxicity to healthy tissues. The PSs can either be conjugated to or encapsulated in clinically‐approved organic NPs^[^ ^311^ ^]^ and biomolecules attached to the surface may then enable targeted delivery. Examples of such formulations include PLGA NPs and liposomes, while Au NPs and Si NPs have also been investigated preclinically.^[^ ^312^, ^313^, ^314^, ^315^ ^]^

#### 6.2.1 PLGA

**Figure 14:**
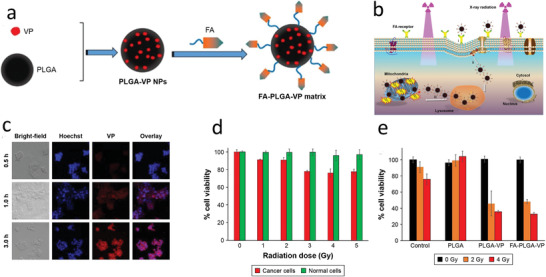
a) Formulation of FA‐PLGA‐VP NPs. b) Schematic of cell uptake via FR‐mediated endocytosis. c) Confocal image showing the uptake of FA‐PLGA‐VP by HCT 116 cell lines at different incubation time. d) HCT116 and normal (CCD 841 CoN) cell kill for different X‐ray doses using the targeted NPs. e) X‐PDT cell kill for different constructs and radiation doses. Reproduced with permission.^[^ ^392^ ^]^ Copyright 2018, Dove Medical Press Ltd.

Polymer NP PLGA NPs are widely used for drug delivery, bioimaging, and diagnostics^[^ ^300^, ^316^, ^317^ ^]^ diagnostics. PLGA can encapsulate hydrophobic drugs and the NPs are themselves hydrophilic, which facilitates biodistribution.^[^ ^318^, ^319^, ^320^ ^]^ The biodegradability of PLGA is due to hydrolysis of its ester linkages in the presence of water, which produces glycolic and lactic acids that can be safely metabolized.^[^ ^321^ ^]^ In addition, the surface of PLGA NPs can be modified with, e.g., PEG, which can further improve biocompatibility and provide colloidal stability.^[^ ^322^, ^323^, ^324^ ^]^ PLGA NPs have been conjugated with folic acid (FA) to enhance specificity to HCT116 colorectal cancer cells (**Figure** **14**a).^[^ ^392^ ^]^ The conjugation and encapsulation were confirmed by absorption spectra (Figure 14b), and it was found that VP is stable inside the NPs for several hours. X‐PDT with these NPs showed efficient tumor cell killing (67% compared with 26% for 4 Gy X‐ray dose only).

#### 6.2.2 Liposomes

Liposomes have also been used to deliver encapsulated PSs in X‐PDT. These are artificial spherical vesicles with an enclosed phospholipid bilayer structure NPs.^[^ ^325^ ^]^ The aqueous core allows the encapsulation of hydrophilic drugs, while the lipophilic bilayer allows encapsulation of hydrophobic drugs.^[^ ^326^ ^]^ Liposomes have the potential to be engineered to release payloads upon external triggering and thermosensitive‐, pH‐, magnetic‐, and photosensitive liposomes have been reported.^[^ ^327^, ^328^ ^]^ Liposomes can also be modified with targeting agents such as folic acid, transferrin or antibodies, to increase their specificity to cancer cells and minimize off‐target effects.^[^ ^312^ ^]^ Different types of PS, such as porphyrins, phthalocyanine, and VP have been encapsulated in liposomes and used in conventional visible‐light‐mediated PDT in both preclinical and clinical settings.^[^ ^326^, ^329^, ^330^, ^331^, ^332^, ^333^, ^334^, ^335^, ^336^, ^337^, ^338^, ^339^ ^]^ It has also been demonstrated that X‐rays can directly activate VP loaded inside liposomes to generate ROS;^[^ ^282^ ^]^ a similar concept using linoleic acid hydroperoxide molecule has also been reported.^[^ ^340^ ^]^

**Figure 15:**
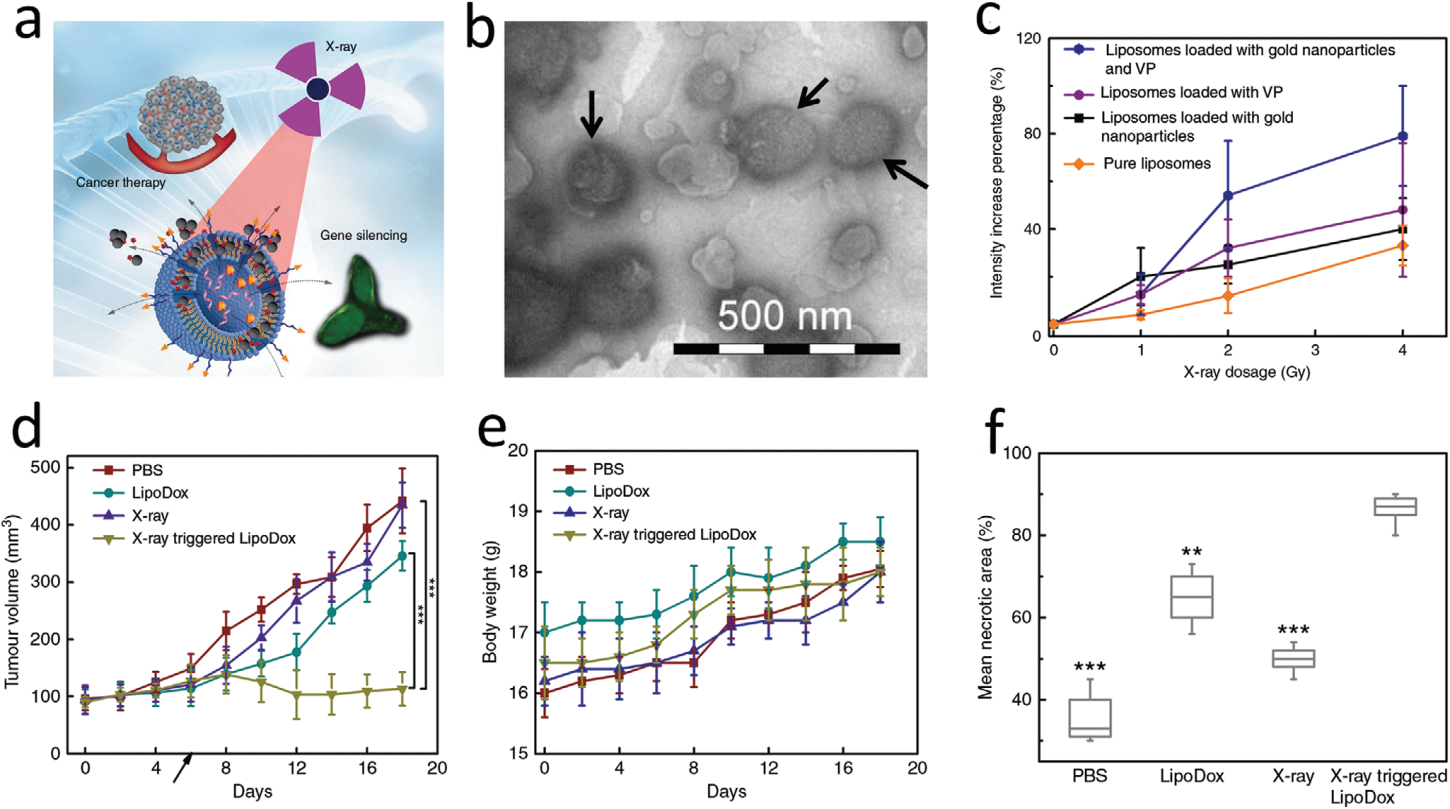
a) Illustration of X‐ray assisted gene silencing and cancer cell killing. b) TEM image of liposomal NPs incorporating with Au NPs and VP. c) Singlet oxygen generation measured by a fluorescent probe (SOSG) from various liposomal formulations. d,e) Antitumor activity of X‐ray triggered LipoDox in a xenograft model of colorectal cancer as measured by tumors volume d) and body weight e) after various treatments administered at the time indicated by the arrow. f) Mean percent tumor necrosis for various treatments. Reproduced with permission.^[^ ^282^ ^]^ Copyright 2018, Springer Nature.

X‐ray‐activatable liposomes incorporating Au NPs (3–5 nm) and VP in the bilayer and a chemotherapy drug (doxorubicin)) or gene‐silencing oligonucleotide (PACIR antisense) in the core have been described by Deng et al.^[^ ^282^ ^]^ Under 6 MeV X‐ray exposure the VP was activated and generated ^1^O_2_ that destabilized the liposome structure to release the Dox or oligonucleotide in a controllable way. Although the VP is the main source of ROS here, the Au NPs also generate some ROS^[^ ^169^, ^341^, ^342^ ^]^ and contribute to the overall efficacy^[^ ^343^ ^]^ that was demonstrated by the efficiency by gene silencing in vitro and chemotherapy in vivo. This technology is illustrated in **Figure** **15**.

### 6.3 Inorganic Nanoparticles Integrated with Photosensitizers

**Figure 16:**
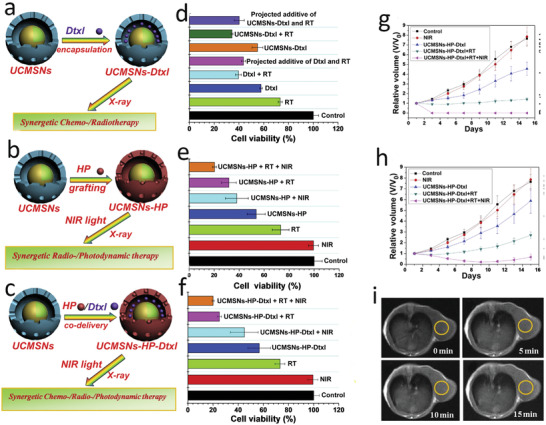
a) Representation and d) in vitro evaluation of synergetic chemo‐/radiotherapy effects on HeLa cells after coincubation with free Dtxl/UCMSNs‐Dtxl. b) Representation and e) in vitro evaluation of synergetic radio‐/photodynamic therapy after coincubation with UCMSNs‐HP. c) Representation and f) in vitro evaluation of synergetic chemo‐/radio‐/photodynamic therapy after coincubation with UCMSNs‐HP‐Dtxl. g) Tumor growth following chemo‐/radio‐/photodynamic therapy on 4T1 tumor‐bearing mice after intratumoral injection of UCMSNs‐HP‐Dtxl. h) Tumor growth after intravenous injection of UCMSNs‐HP‐Dtxl. i) In vivo T1eMRI images of a 4T1‐tumor bearing mouse after intravenous injection of UCMSNs at designated time points: (a) 0 min, (b) 5 min, (c) 10 min, and (d) 15 min. Reproduced with permission.^[^ ^355^ ^]^ Copyright 2014, Biomaterials.

Inorganic NPs other than nanoscintillators have also been explored for X‐PDT, including complex nanoformulations that can be used in combination with ionizing radiation, either as a radiosensitizer or to transfer energy to a PS or as a PS per se. These various formulations can also be functionalized with tumor‐targeting moieties. Mesoporous silica NPs (MSNs) and upconverting NPs (UCNPs), are also being investigated as potential candidates for carrying PSs.^[^ ^344^ ^]^ Their surface functionalities and biocompatibilities allow controlled drug release and targeted drug delivery.^[^ ^345^, ^346^, ^347^, ^348^ ^]^ Several reports discuss silica as an efficient carrier for PDT drugs such as RB, PpIX, and Ce6^[^ ^349^, ^350^, ^351^, ^352^, ^353^, ^354^ ^]^ and some of these were used in combination with scintillating NPs.^[^ ^27^, ^293^, ^297^ ^]^ For example, an effective multifunctional nanotheranostic system was constructed using Gd‐UCNPs (NaYF4:Yb (18%)/Er(2%)/Tm (1%)) as the core and mesoporous silica as a shell (UCMSN). A PS, hematoporphyrin (HP), and a chemotherapy drug, docetaxel (Dtxl) were loaded to this NP.^[^ ^355^ ^]^ The NPs could be triggered either by ionizing radiation or by NIR through energy transfer from the UCNPs. **Figure** **16**a,d demonstrates the synergetic chemo‐radiotherapy effects on HeLa cells incubated with UCMSN‐Dtxl. Dtxl itself is a clinically approved chemo/radiosensitizer drug but it has poor solubility that has limited its efficacy. Here, the UCMSN‐Dtxl showed enhanced therapeutic effects compared to free Dtxl. Similarly, HP loaded into UCMSN by covalent conjugation led to decreased tumor cell viability after treatment with radiotherapy and NIR which was (20%) was lower than with radiotherapy (32%) or NIR alone (38%) (Figure 16b,e). However, codelivery of Dtxl and HP was not more effective than individual delivery (Figure 16c,f). Figure 16g,h shows the in vivo results in a subcutaneous 4T1 breast cancer model. This nanoconstruct can also be used as an MRI contrast agent (Figure 16i).

**Table** **4** presents representative examples of all X‐PDT nanomaterials in the literature. Comprehensive summaries have been reported elsewhere.^[^ ^39^, ^356^, ^357^, ^358^, ^359^, ^360^ ^]^

**Table 4:** Representative NPs used in X‐PDT

Nanoparticles (NP)	Photosensitizer (PS)	Role of NP in X‐PDT	Radiation conditions	Targeted (yes/no)	In vitro/In vivo	Ref.
Au clustoluminogens (AIE‐Au)	Rose Bengal (RB)	Radiosensitization and energy transfer to PS	50 kV, 70 µA, 1 Gy	Yes: *α*_v_ *β*_3_ integrin receptor targeting	In vivo	^[^ ^360^ ^]^
Au	Verteporfin (VP)	Prevention of aggregation of PS and radiosensitization	6 MV, 6 Gy	No	In vitro	^[^ ^283^ ^]^
Au@SNs	PPIX	Radiosensitization	6 MV, 2 Gy	No	In vivo	^[^ ^361^ ^]^
BiOI@Bi_2_S_3_@BSA (SHNP)	–	Radiosensitization	50 kV, 80 µA, 6 Gy	No	In vivo	^[^ ^202^ ^]^
CLC/SPIO micelles	Ce6	Radiosensitization	6 MV, 2 Gy	Yes: Folic acid targeted	In vivo	^[^ ^362^ ^]^
Cu–Cy	Cu–Cy	Radiosensitization	90 kV, 30 mA	No	In vivo	^[^ ^363^ ^]^
GQDs	PPIX	Radiosensitization and energy transfer to PS	6 MV, 2 Gy	Yes‐Bacteria‐targeted	Other	^[^ ^364^ ^]^
Hf_6_O_4_(OH)_4_(HCO_2_)_6_ SBUs	Ir[bpy ppy)_2_]^+^ and [Ru(bpy)^3^]^2+^	Energy transfer to PS	120 kV, 20 mA	No	In vitro	^[^ ^351^ ^]^
Hf‐DBB‐Ru	DBB‐Ru Ru(bpy)_3_^2+^	Energy transfer to PS	225 kV, 13 mA 1 Gy	Mitochondrial targeting	In vivo	^[^ ^41^ ^]^
(*n*‐Bu_4_N)_2_[Mo_6_I_8_(OOC‐1‐adamantane)_6_]	(*n*‐Bu_4_N)_2_[Mo_6_I_8_(OOC‐1‐adamantane)_6_]	Radiosensitization/energy transfer to PS	40 kV, 15 mA, dose not specified	Nontargeted	In vivo	^[^ ^365^ ^]^
TiO_2_:C	–	Radiosensitization	80 kV 10 mA 0.08 Gy min^−1^	Nontargeted	In vitro	^[^ ^366^ ^]^
TiO_2_:Ce	–	Radiosensitization	80 KV, 10 mA, 0.08 Gy min^−1^ for 100 s	Nontargeted	In vivo	^[^ ^367^ ^]^
W IV doped ZnGa_2_O_4_:Cr	ZnPcS_4_	Energy transfer to PS	50 kV, 60 µA, 0.18 Gy	Nontargeted	In vivo	^[^ ^368^ ^]^
ZnO	MTCP and CuMTCP	Energy transfer to PS	70 kV, 8 mA, 0.94 Gy for 30 s	Nontargeted	In vitro	^[^ ^369^ ^]^

## 7 Clarification of Terminology Used in the Field

The boundaries of terms “radiosensitization,” “X‐PDT,” and “radiodynamic therapy” are somewhat fluid in the subject literature, and we feel that they are worth discussing to avoid unnecessary disputes.

With respect to radiosensitization, according to Gallez^[^ ^370^ ^]^ it is “a physical, chemical, or pharmacological intervention that increases the lethal effects of radiation when administered in conjunction with it.” Gallez describes only chemical and biologic response modifiers. The NCI Dictionary of Cancer Terms^[^ ^371^ ^]^ states that “radiosensitization is the use of a drug that makes tumor cells more sensitive to radiation therapy.” The Medical Dictionary^[^ ^372^ ^]^ goes even further and states that radiosensitizer is “a chemotherapeutic agent used to enhance the effect of radiation therapy.” In this work we adopt a definition that radiosensitization is a process of interference with cells to make cells more sensitive to radiation. This interference with cellular processes may take the form of increasing the amount of ROS generated from a defined radiation dose or making cells more vulnerable, by interfering with molecular targets in cells,^[^ ^373^ ^]^ for example, by disarming cellular antioxidant defences or exposing cells to radiant heat, while the amount of ROS generated by radiation remain unaffected. Clinically used radiosensitizers (Section 3.5) belong to the latter category.

The term X‐PDT refers to the method first introduced by Chen and Zhang in 2006^[^ ^38^ ^]^ who combined a nanomaterial with X‐rays resulting in generation of ROS. This process is also referred to as X‐ray mediated PDT or (rarely) PDT‐X. X‐PDT is defined as the use of X‐rays instead of UV–vis light in combination with PS where the use of X‐rays enables deeper penetration into tissue. PS is an agent that generates ROS upon interaction with photons. According to NCI Dictionary of Cancer Terms photosensitizer is a drug (also called photosensitizing agent) used in photodynamic therapy. When absorbed by cancer cells and exposed to light, this drug becomes active and kills the cancer cells. Accordingly, in this work we refer to X‐PDT as combining materials (molecular, nanomaterials or their combinations) with X‐rays to generate ROS above and beyond what would be generated by the same dose of X rays in the tissue. We note that the literature in the area of X‐PDT is predominantly focussed on designing various nanomaterials which tend to play an active role to generate ROS and not just act as carriers. We also emphasize that, according to our definition, X‐PDT is a special form of radiosensitization that is drawing on mechanisms from physics, chemistry, and materials engineering approaches rather than on mechanisms in cell biology. Furthermore, all X‐PDT materials discussed here are radiosensitizers.

The term radiodynamic therapy is used less frequently,^[^ ^42^ ^]^ and it typically refers to combining molecular PSs (or equivalent) with X‐rays. However, it is also used when molecular PSs are encapsulated in NP carriers (such as polymer carriers) which do not—on their own—generate additional reactive oxygen species when exposed to X‐ray radiation.

## 8 Future Directions for Materials Science in X‐PDT

Free radical generation by NPs interacting with X‐rays in conditions compatible with clinical radiotherapy offers a potential new option for cancer therapy. Generated in amounts over and beyond those produced by radiation alone, this approach offers defined advantages. Most notably, X‐PDT allows to overcome the depth limitation of conventional photodynamic therapy using an external light source.^[^ ^28^ ^]^ By inducing free radicals at sub‐clinical radiation doses, X‐PDT could provide an effective treatment of deep‐seated and larger solid tumors. The use of NPs allows these free radicals to act alone, or in combination with other codelivered agents (oxygen sources, therapeutic drugs, genes, etc.), and agents that may be modified or released by the free radicals, offering opportunities for new modes of action, such as precisely synchronous delivery of drugs and radiation, with the potential for synergistic action and multimodality. This is because nanomaterials can be endowed with a wide range of sophisticated functionalities. Opportunities such as these have stimulated many groups to publish in excess of 900 papers in the area which were cited over 23 000 times. However, we feel that the research and downstream clinical opportunities are much wider and deeper than what is pursued currently. With that in mind, this review discusses and indicates areas for future research opportunities and potential clinical translation. It is widely recognized that the X‐PDT technology sits at the intersection of clinical radiation therapy, photomedicine and nanotechnology. It also intersects with tumor biology and pharmacology that have yet to be fully exploited to maximize the efficacy and safety of X‐PDT. For example, mainstream megavoltage X‐rays have been mainly used in X‐PDT to date, with limited exploration of radioisotopes. However, high‐LET radiation such as obtained with proton accelerators is also emerging and could open up new avenues for X‐PDT. Complementing this, there are vast areas of nanotechnology that currently seem unrelated to medical applications, such as photocatalysis, solar energy conversion, clean energy, environmental remediation, that have developed sophisticated solutions to engineer NPs and their surfaces to optimize generation of free radicals using light. Some of these approaches might be effective when light is replaced by ionizing radiation, and examples have been given in Section 6.

**Figure 17:**
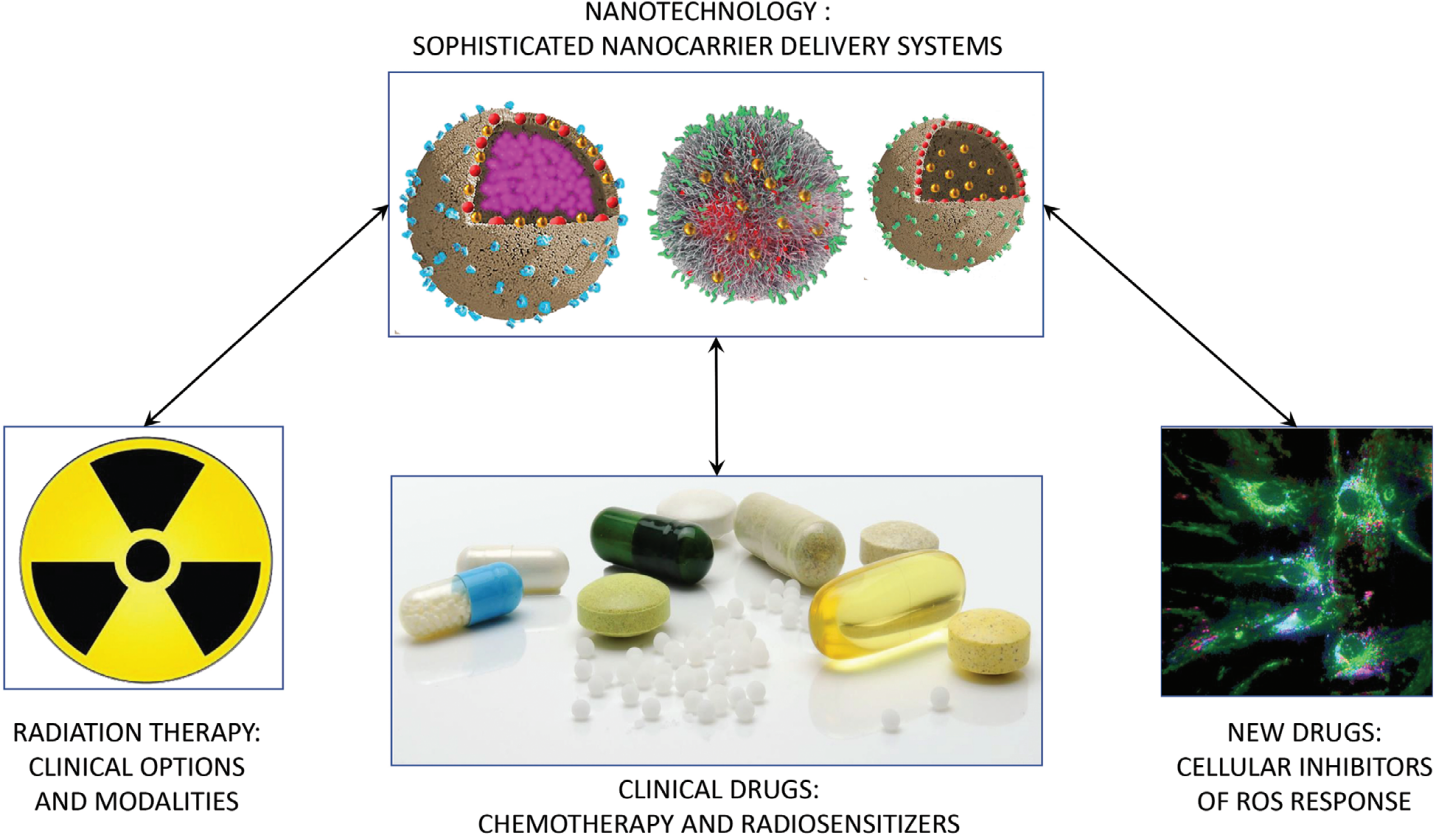
Promising future directions for materials science in X‐PDT.

Furthermore, nanotechnology research has introduced many options for sophisticated drug delivery, that can be applicable to X‐PDT and which offer unique advantages, including those highlighted in Section 6.2. However, the field is only beginning to explore the opportunities in relation to the agents and drugs that can be codelivered with the free radicals generated during X‐PDT. There are also similar opportunities to incorporate inhibitors of cellular processes driving the cellular response to radiation into the X‐PDT nanomaterials, as highlighted in Section 3.4, Even more importantly, there is a possibility to use NP formulations to deliver defined “cocktails” of clinically approved therapeutics, including chemotherapy drugs and radiosensitizers. As addressed in Sections 6.2.1 and 6.2.2, it is possible to use nanomaterial carriers built from clinically approved materials and to use clinically approved PSs for X‐PDT, which would accelerate clinical translation. Importantly, some of these carriers (Section 6.2.2) offer unique opportunities for spatial and temporal control of drug delivery, for example, the patented technology for X‐ray triggering of liposomes.^[^ ^282^, ^374^ ^]^ These options are illustrated in **Figure** **17**.

## 9 Potential and Challenges in Clinical Translation of X‐PDT

Successful^[^ ^169^ ^]^ development of X‐PDT, including the use of nanomaterials incorporating PSs and other clinical drugs and agents, has high potential for clinical translation, particularly in oncology. The opportunities will exploit many of the fundamental advantages and capabilities of the novel nanomaterials, as discussed in this paper. This clinical translation falls into two broad categories that, while they may share aspects of the underlying science, would be quite distinct in their clinical utilization: a) X‐PDT as a stand‐alone therapeutic modality where X‐rays stimulate a systemically delivered traditional PS drug, and b) the use of NP, NP PS or NP‐PS‐drug formulations (at the focus of this review) as radiation sensitizers for conventional fractionated radiation therapy. In both cases the methods of external‐beam radiotherapy would be employed while, analogously, radioactive sources could be used as the energy source, either using systemically administered radionuclides as in radioimmunotherapy or implanted solid sources as in brachytherapy.

The stand‐alone approach would, ideally, involve the use of a single X‐ray exposure, or small number of fractions, with a total X‐ray dose that is much smaller than that used for conventional radiotherapy, with the intent to destroy the tumor or to down‐stage the disease for subsequent additional therapies such as surgical resection. As already indicated, compared with conventional PDT using external (laser, LED) light sources, this would have the advantage of enabling treatment of deep‐seated and larger tumors without the complexity of fiber‐optic or endoscopic light delivery.^[^ ^87^ ^]^ In principle, the modality could be used either for primary solid tumors or for more disseminated disease, including types of metastatic cancer which are technically very difficult or impossible to treat using external light sources. X‐PDT as a stand‐alone modality can also be expected to preserve some of the main biological advantages of PDT,^[^ ^140^ ^]^ that include low systemic toxicity, good healing of normal tissues and immune upregulation that may contribute significantly to the treatment efficacy.^[^ ^375^ ^]^ Another advantage of PDT is the ability to repeat the treatment as often as required to sustain tumor control: however, this would be impaired in X‐PDT, as the X‐ray exposure would be cumulative and limiting.

In the second approach, the NP‐PS‐drug formulation would be used to increase the tumor cell kill during conventional multifractionated radiotherapy or brachytherapy, i.e., as a novel form of radiosensitization.^[^ ^39^, ^72^ ^]^ Recognizing that collateral damage to normal tissues in the radiation field is often dose‐limiting in radiation therapy, tumor‐selective radiosensitization^[^ ^12^, ^376^ ^]^ enables either i) increased tumor response using the same dose or ii) reduced normal tissue damage and resulting complications by using the same dose while maintaining the same tumor response.

In addition to opportunities, there are many challenges in translating X‐PDT with these various NPs and formulations into clinical oncology practice, including those listed in **Table** **5**. First and most critical in stand‐alone X‐PDT is to achieve a clinically impactful efficacy at an X‐ray dose that is well tolerated level by normal tissues within the radiation field, say <10 Gy compared to a typical fractionated radiotherapy dose of 50–70 Gy. It should be noted that a very small amount of energy is required to cause DNA strand breaks with X‐rays, leading to proliferative cell death, whereas light‐activated PDT normally relies primarily on somatic cell death mediated by damage to membrane structures.^[^ ^375^ ^]^ For example, a 50 Gy X‐ray dose corresponds to an absorbed energy of 0.05 J g^−1^ of tissue, whereas a typical incident surface light irradiance in conventional PDT is ≈100 J cm^‐2^ so that, assuming the light is absorbed within 1 cm tissue thickness, the energy absorbed is ≈100 J g^−1^, i.e., 3 or 4 orders‐of‐magnitude greater than in radiotherapy. Hence, X‐ray PDT will likely require some form of biophysical or biological enhancement or amplification to achieve comparable efficacy. Overcoming the energy barrier by targeting the NPs to the cell nucleus^[^ ^113^, ^114^, ^115^ ^]^ could provide the necessary amplification, as has been shown with PDT itself.^[^ ^377^ ^]^ Molecular targeting of cancer and nuclear targeting would also facilitate using systemic administration of the NPs, avoiding the need for intratumoral injection to achieve very high local concentration that has been reported in some studies of X‐PDT (e.g., as in Figure 6 ^[^ ^198^ ^]^) but is problematic to apply clinically. Using X‐PDT as a novel form of radiosensitization for conventional radiotherapy also circumvents this energy problem, since the modest increase in tumor cell kill per X‐ray dose fraction reported in several X‐PDT studies (e.g., as in Figure 14) could still result in significantly increased treatment efficacy:, e.g., 20% higher cell kill per 2 Gy fraction translates to >2 logs of additional tumor cell death after 30 fractions.

**Table 5:** Challenges in clinical translation of X‐PDT

	CHALLENGE
Fundamental	Adequate cell kills at well‐tolerated X‐ray dose with systemic nanomaterials administration Adequate nanoparticle tumor‐to‐normal selectivity and ensuring tumor specificity. Acceptable toxicities: systemic, skin/ocular phototoxicity, unfavorable pharmacological profiles
Practical	Overcoming biological barriers in nanoparticle delivery to cancer Optimal time between nanoparticle administration and X‐irradiation, accounting for cumulative uptake and clearance Treatment planning incorporating nanomaterials uptake in individual patients : dose algorithms and software : nanoparticle uptake measurements in tumor and normal tissues
Clinical	Trial(s) design Adoption and integration into clinical practice: efficacy versus risks, cost, and complexity
Regulatory	Controllability and manufacturing reproducibility and variations of nanoparticle formulations (nanoparticle heterogeneity, aggregation, and dissociation) Approvals for combination nanoparticle‐radiation treatments: safety and efficacy
Commercial	Feasibility and ownership of IP protection of nanoparticle formulations and X‐PDT technologies Cost‐benefit as stand‐alone modality or as radiation sensitizer

A second major challenge is to achieve adequate tumor specificity of the NP complement the spatial targeting of the X‐ray beams. For example, in the radiosensitization case above, if the tumor‐to‐normal tissue uptake is only 2:1, then the differential increase in tumor cell kill after 30 fractions drops to <20‐fold (1.2^30^/1.1^30^), assuming a linear dependence of this effect on NP concentration in the cells. Active targeting of nanomaterials to tumors, beyond the intrinsic EPR effect, has been discussed above^[^ ^18^, ^19^ ^]^ and is the focus of other reviews.^[^ ^378^ ^]^ These and other authors review^[^ ^11^, ^43^, ^379^, ^380^, ^381^ ^]^ and discuss biological barriers in targeted NP delivery to tumors such as half‐life of blood circulation, or opsonization, and opportunities, e.g., engineering of tumor‐associated macrophages to act as a reservoir of nanotherapeutics from which the drug payload is gradually released. From the translational perspective, there is nothing particular in targeting nanomaterials for X‐PDT, except perhaps if additional subcellular organelle targeting is required. Importantly, efficient and selective tumor targeting also enables the total dose of NPs administered to the patient to be reduced, which addresses the third challenge of their potential toxicities. Again, the general topic of NP toxicity has been reviewed in depth elsewhere^[^ ^382^ ^]^ and the same general issues arise, due both to the component organic and inorganic materials (including, e.g., scintillation nanocrystals if used) and to the nanoparticulate nature itself. Examples of toxicity management of nanomaterials including inorganic quantum dots is reviewed by Corbo and co‐workers,^[^ ^383^, ^384^ ^]^ while Liu et al. and Nel et al.^[^ ^385^, ^386^ ^]^ discuss regulatory challenges for toxicity testing of nanomaterials designed for clinical applications. Additional factors in NP‐mediated X‐PDT are the potential of damage to skin or the eyes from light exposure of the PS component, which has sometimes been a limiting toxicity in conventional PDT with some PSs,^[^ ^140^ ^]^ which has not been examined in X‐PDT studies to our knowledge.

At the practical level, particularly as a stand‐alone treatment, X‐PDT in patients will require a treatment planning platform, most likely implemented by extending current radiotherapy systems^[^ ^387^ ^]^ to incorporate information on the NP concentration in tumor and normal tissues within the radiation field. In turn, this raises the challenge of how the NP uptake will be determined in individual patients, which is not usually a significant issue in diagnostic applications but will be critical for X‐PDT to be used safely and effectively, at least in stand‐alone mode.

From the clinical perspective, the design of human trials of X‐PDT will combine many of the same challenges as in conventional PDT trials^[^ ^388^ ^]^ as well as those of radiotherapy trials, and will involve NP and X‐ray dose escalation, with corresponding assessment of tumor response and off‐target effects. Ultimately, clinical X‐PDT is most likely to fall under radiation oncology in order to access the existing equipment infrastructure and expertise, whereas conventional PDT has mostly been used by surgeons, endoscopists, dermatologists, and ophthalmologists. Corresponding changes in education and training of radiation oncologists, radiation physicists, and technologists will be required, as well as the infrastructure to administer the NPs safely. Optimizing the timing between NP administration and X‐irradiation will be challenging and, where multiple X‐ray fractions are employed, the clearance rate from the tumor and normal tissues will determine the frequency of multiple NP injections, with corresponding concern for potential cumulative toxicities. This would be less of an issue if X‐PDT were to be delivered in a small number of fractions, analogous to stereotactic body radiation therapy, SBRT.^[^ ^389^ ^]^ Ultimately, the efficacy of X‐PDT will need to be high enough to justify its added costs and logistical complexity for it to be adopted into radiation oncology practice. As with any other new medical technology, the cost‐benefit will need to be sufficiently favorable for it to be commercially viable.^[^ ^390^ ^]^

If these challenges can be overcome, then X‐PDT offers exciting opportunities for research and clinical innovation that could make a real difference in cancer control. In parallel, there are likely opportunities also outside oncology, just as PDT itself has been investigated and applied to address a range of unmet clinical needs.^[^ ^391^ ^]^

## Conflict of Interest

The authors declare no conflict of interest.
